# Neuroscience from the comfort of your home: Repeated, self-administered wireless dry EEG measures brain function with high fidelity

**DOI:** 10.3389/fdgth.2022.944753

**Published:** 2022-07-29

**Authors:** Florentine M. Barbey, Francesca R. Farina, Alison R. Buick, Lena Danyeli, John F. Dyer, Md. Nurul Islam, Marina Krylova, Brian Murphy, Hugh Nolan, Laura M. Rueda-Delgado, Martin Walter, Robert Whelan

**Affiliations:** ^1^School of Psychology, Trinity College Dublin, Dublin, Ireland; ^2^Cumulus Neuroscience Ltd., Dublin, Ireland; ^3^Trinity College Institute of Neuroscience, Dublin, Ireland; ^4^Cumulus Neuroscience Ltd., Belfast, United Kingdom; ^5^Department of Psychiatry and Psychotherapy, Jena University Hospital, Jena, Germany; ^6^Department of Behavioral Neurology, Leibniz Institute for Neurobiology, Magdeburg, Germany; ^7^Clinical Affective Neuroimaging Laboratory, Magdeburg, Germany; ^8^Medical Physics Group, Institute of Diagnostic and Interventional Radiology, Jena University Hospital, Jena, Germany; ^9^Trinity Centre for Biomedical Engineering, Trinity College Dublin, Dublin, Ireland; ^10^Department of Psychiatry and Psychotherapy, University of Tübingen, Tübingen, Germany; ^11^Medical Faculty, Otto von Guericke University of Magdeburg, Magdeburg, Germany

**Keywords:** electroencephalography, longitudinal, cognition, gamification, humans, dry electroencephalography, Standard Measurement Error, signal quality

## Abstract

Recent advances have enabled the creation of wireless, “dry” electroencephalography (EEG) recording systems, and easy-to-use engaging tasks, that can be operated repeatedly by naïve users, unsupervised in the home. Here, we evaluated the validity of dry-EEG, cognitive task gamification, and unsupervised home-based recordings used in combination. Two separate cohorts of participants—older and younger adults—collected data at home over several weeks using a wireless dry EEG system interfaced with a tablet for task presentation. Older adults (*n* = 50; 25 females; mean age = 67.8 years) collected data over a 6-week period. Younger male adults (*n* = 30; mean age = 25.6 years) collected data over a 4-week period. All participants were asked to complete gamified versions of a visual Oddball task and Flanker task 5–7 days per week. Usability of the EEG system was evaluated via participant adherence, percentage of sessions successfully completed, and quantitative feedback using the System Usability Scale. In total, 1,449 EEG sessions from older adults (mean = 28.9; SD = 6.64) and 684 sessions from younger adults (mean = 22.87; SD = 1.92) were collected. Older adults successfully completed 93% of sessions requested and reported a mean usability score of 84.5. Younger adults successfully completed 96% of sessions and reported a mean usability score of 88.3. Characteristic event-related potential (ERP) components—the P300 and error-related negativity—were observed in the Oddball and Flanker tasks, respectively. Using a conservative threshold for inclusion of artifact-free data, 50% of trials were rejected per at-home session. Aggregation of ERPs across sessions (2–4, depending on task) resulted in grand average signal quality with similar Standard Measurement Error values to those of single-session wet EEG data collected by experts in a laboratory setting from a young adult sample. Our results indicate that easy-to-use task-driven EEG can enable large-scale investigations in cognitive neuroscience. In future, this approach may be useful in clinical applications such as screening and tracking of treatment response.

## Introduction

Reliable, objective assessment of brain function is a crucial part of the evidence base for cognitive neuroscience and for the characterization of neurodegenerative, neurodevelopmental, and neuropsychiatric disorders. Many external factors can affect measurements of cognitive functioning, including mood, stress, and energy levels (1–8). As such, measuring brain activity at any single time point may not accurately reflect a person's typical neurocognitive profile. Furthermore, individual and group-level estimates of neurophysiological phenomena may improve with aggregation over larger samples—the same principle that underlies the use of repeated samples in a single behavioral or neuroimaging experimental session. Repeated recording of brain activity—over many days—may therefore allow for the identification of more reliable metrics of brain function and cognitive performance, that account for day-to-day variability (9–11).

EEG is the longest-established and most validated technology for sampling brain function (12), with a deep and broad literature establishing key neurophysiological measures of specific cognitive domains including executive function, memory, sensory processing and motor planning/execution (13–16). Recent progress in EEG technology presents additional opportunities for identifying reliable neurocognitive metrics and biomarkers (9–11, 17, 18). For example, advances in sensor development, signal processing, and cloud-based technologies have led to the creation of wireless, dry EEG recording systems that can be operated remotely and repeatedly by naïve users (11, 18–21). As a result, EEG data can now be collected frequently by research participants themselves over days, weeks, or months.

To date, evaluation of dry EEG has consisted of direct comparisons with conventional wet (i.e., electrolytic gel or water-based) technology or test-retest assessments in controlled environments (9, 10, 18, 19, 22–24). While these studies demonstrated that dry EEG can perform similarly to wet EEG in controlled settings, they did not address the question of EEG feasibility and variability in ecologically valid environments. In particular, the usability of self-administered EEG as well as the amount of data (i.e. trials, sessions) required from at-home settings when data is self-recorded is unclear.

One challenge with repeated neurocognitive testing protocols is maintaining participant engagement over extended periods (25, 26), as has been investigated more extensively in purely behavioral testing (27–32). Neurocognitive tasks used in EEG research often require participants to respond to simple geometric stimuli presented on a uniform background, with deliberately little variation among trials over several minutes. Maintaining adherence to these tasks in the home environment is challenging (28, 33, 34), leading to elevated attrition rates that reduce sample sizes, waste participant effort, and bias results (35, 36). Gamification of laboratory paradigms—adding game-like features (points, graphics, levels, storyline, etc.,)—may address these problems by keeping participants engaged for longer (26, 37, 38). For example, a gamified behavioral spatial memory task administered longitudinally was more effective at classifying high-risk Alzheimer's disease (AD) participants than traditional neuropsychological episodic memory tests (39).

The Oddball and Flanker tasks are two of the most widely used neurocognitive tasks in EEG research that produce characteristic event-related potential (ERP) components, making them ideal candidates for gamification. These tasks are sensitive to individual differences in attentional allocation, processing speed (40), and error awareness (41, 42), as well as group differences in executive functioning (43) and decision making (40, 44). The Oddball task elicits the P300 ERP—a positive voltage deflection over centro-parietal areas approximately 300 ms post-stimulus, which is thought to reflect decision making, attention and working memory processes (40, 45–48). The Flanker task elicits the error-related negativity (ERN)—a negative voltage deflection observed fronto-centrally after an erroneous response, which is thought to reflect adaptive response (43, 49, 50) and attentional control processes (50). The ERN is followed by a posterior positive rebound, known as the Error Positivity (Pe), which is linked to conscious error recognition (51). The ERN and Pe are typically computed by subtracting response-locked ERPs extracted from Correct trials to response-locked ERPs extracted from incorrect trials. Response-locked ERPs extracted from Correct trials consist of a positive deflection between 0 to 50 ms after a correct answer while Error trials consist of a negative deflection peaking 50–100 ms after an erroneous response. In previous work, we demonstrated the face validity of gamified visual oddball and flanker EEG tasks (52). Our analyses demonstrated that ERPs evoked from gamified tasks yield signals similar in morphology and topography to those evoked by standard neurocognitive tasks used in EEG research. Here we aim to further that validation by looking at the variability of signals over repeated at-home sessions.

A key question when designing ERP studies is the strategy taken to balance the quality and quantity of data recorded, as this will have an effect on the statistical power of experimental analyses. In laboratory environments it is straightforward to control signal quality, but noise and artifacts still occur, meaning choices must be made in how participants and/or individual behavioral trials (and the EEG epochs they correspond to) are included or excluded—which may be more liberal (lower quality, higher quantity) or conservative (higher quality, lower quantity). The desire to record more trials, over a longer task duration, must be balanced against considerations of a fair burden on users, and the fact that participants may become fatigued and/or disengaged over time. In a remote study, self-administered by non-experts, there are additional challenges to quality (which we may expect to be more variable than in controlled laboratory settings), but opportunities for data quantity, as it is feasible to ask participants to split a larger amount of task-driven behavioral interaction over a number of days. In this paper we explore this question in the context of gamified tasks that should encourage repeated task engagement, and with older and younger user groups for which usability and familiarity with technology may be an issue.

Here, we conducted *post-hoc* analyses of two pre-existing datasets collected for other purposes. Longitudinal dry EEG data were collected from two separate cohorts: older adults aged 55+ years over 6 weeks (53), and younger adults aged 18–35 over 4 weeks (54). In the younger adult study, a pharmacological challenge with ketamine was used in a cross-over design, but the EEG data reported here was from the non-ketamine control condition only. Participants were asked to complete gamified Oddball and Flanker tasks 5–7 times per week while EEG was simultaneously recorded. We sought to evaluate if dry EEG recorded in the home by unsupervised participants over multiple sessions could yield datasets of the same aggregate quality as published results derived from a single wet EEG session recorded in a controlled environment. We evaluated dry EEG signal quality and usability by cohort (older vs. younger adults) and by gamified task (Oddball vs. Flanker). We expected that, when comparing equal numbers of trials, the signal quality of dry EEG would be lower than that of wet EEG; however, we predicted that similar signal quality could be achieved by aggregating dry EEG data across multiple sessions from each participant. To our knowledge, no published study has quantified the variability of self-administered EEG recorded remotely in younger and older population *via* gamified neurocognitive tasks.

## Methods

### Participants

#### Younger adult cohort

These data originated in a pharmacological challenge study investigating the acute and persisting effects of racemic ketamine (54). Participants were recruited either *via* public announcements or from an existing database of participants who took part in previous studies of the *Clinical Affective Neuroimaging Laboratory* (CANLAB) & Leibniz Institute for Neurobiology, Magdeburg, Germany. Right-handed male participants aged 18–55 years were included in the study. Exclusion criteria were a current or lifetime major psychiatric disorder, including substance or alcohol dependence or abuse, according to DSM-IV; family history of psychiatric disorders as assessed by a demographic questionnaire; and neurological or physical constraints or severe illnesses as evaluated by a study physician during screening. Only males were recruited in this study to reduce variability in the sample. Further exclusion criteria were technological barriers to completing the assessment at home (e.g., lack of Wi-Fi connection) and color-blindness. Participants were compensated with €500 (~US$625), which participants received after their involvement in the study, but that amount was not pro-rated to session-wise adherence. This study was approved by the Institutional Review Board of the Otto-von-Guericke University Magdeburg, Germany, and informed consent was obtained from all participants.

#### Older adult cohort

The data analyzed here were collected as part of a larger study on memory performance in healthy aging. Participants were drawn either from an existing database of older adults who had previously taken part in research studies at Trinity College Dublin, Ireland, or recruited directly from the local community *via* word-of-mouth, newspapers, posters, and Facebook advertisements. Participants were included if they were >55 years old. Exclusion criteria were a current diagnosis of, or taking medications for, any psychiatric or neurological illness; substance abuse; color-blindness; or technological barriers to completing the assessment at home (e.g., lack of Wi-Fi connection). Participants also completed the Wechsler Memory Scale Logical Memory test (WMS-IV) (55), the National Adult Reading Test (NART; a measure of pre-morbid IQ) (56) and the Montreal Cognitive Assessment (MoCA) (57). Participation in the dry EEG portion of the study was contingent on a MoCA score >23 [per Murphy et al. (53)]. All participants were compensated with €20 (~US$25) at the end of the screening session. If they agreed to participate in the at-home part of the study, they received an additional €40 for their participation, which was not contingent on adherence. The study was approved by the ethics committee of the School of Psychology of Trinity College Dublin, Ireland, and informed consent was obtained from all participants.

### Tasks

The gamified tasks are proprietary to the technology provider, and not all details of task mechanisms are in the public domain. We provide the core information about the task structure here, as it relates to the user experience of performing the tasks.

#### Gamified visual oddball task

**Figure 1:**
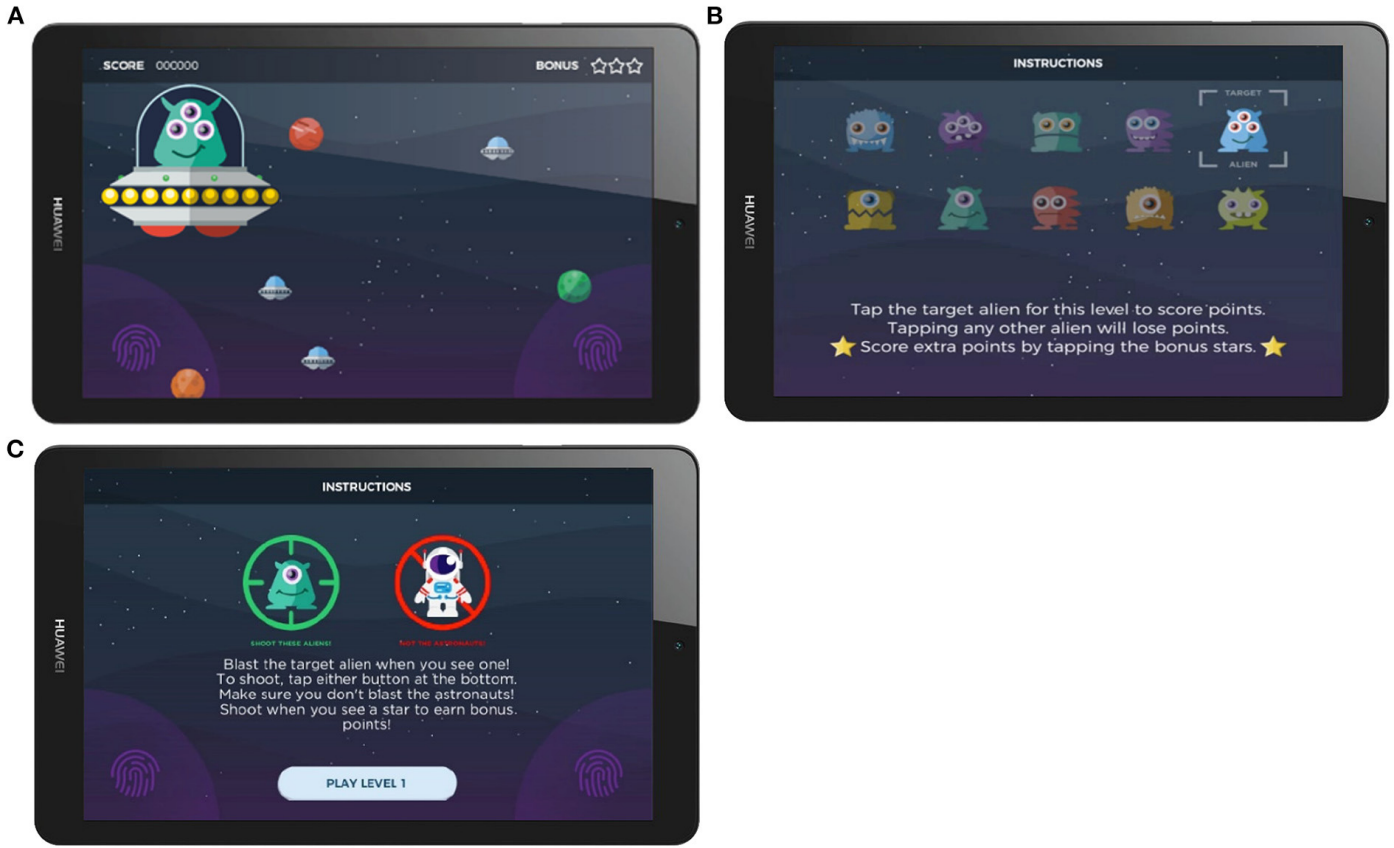
Screenshots of the gamified Oddball task. **(A)** Screenshots of the stimulus presentation, **(B)** Instruction screen in the Older Adult Study, **(C)** Instruction screen in the Younger Adult Study.

Within each session, 30 target stimuli and 70 non-target stimuli were randomly presented across five levels (blocks) of gameplay. In the older adult cohort, targets and non-targets were “aliens” of different colors, expressions, and hairstyles. Responses were made by tapping directly on the stimuli on the tablet screen. In the younger adult cohort (data collected 6 months after the first cohort), the game had been modified in two specific ways: responses were captured by tapping on buttons located on either side of the screen; and non-target stimuli were “astronauts”, not aliens. In both studies, the target aliens changed between levels for variety in gameplay and to encourage an attentive player strategy. During gameplay, the upcoming stimulus locations were highlighted prior to stimulus presentation to encourage user attention, in a manner functionally similar to a fixation cross. Stimuli (e.g., aliens, astronauts) were then visible on-screen for 200 ms. Points were awarded for a correct response and deducted for an incorrect response, in conjunction with auditory feedback. Points awarded for correct responses were mapped to reaction time (faster responses gained more points) to encourage attention and faster response. Each gameplay session lasted approximately 12 min. Screenshots of the gamified Oddball task are presented in Figure 1. Task graphics were designed with a color-blind-safe palette.

#### Gamified adaptive flanker task

**Figure 2:**
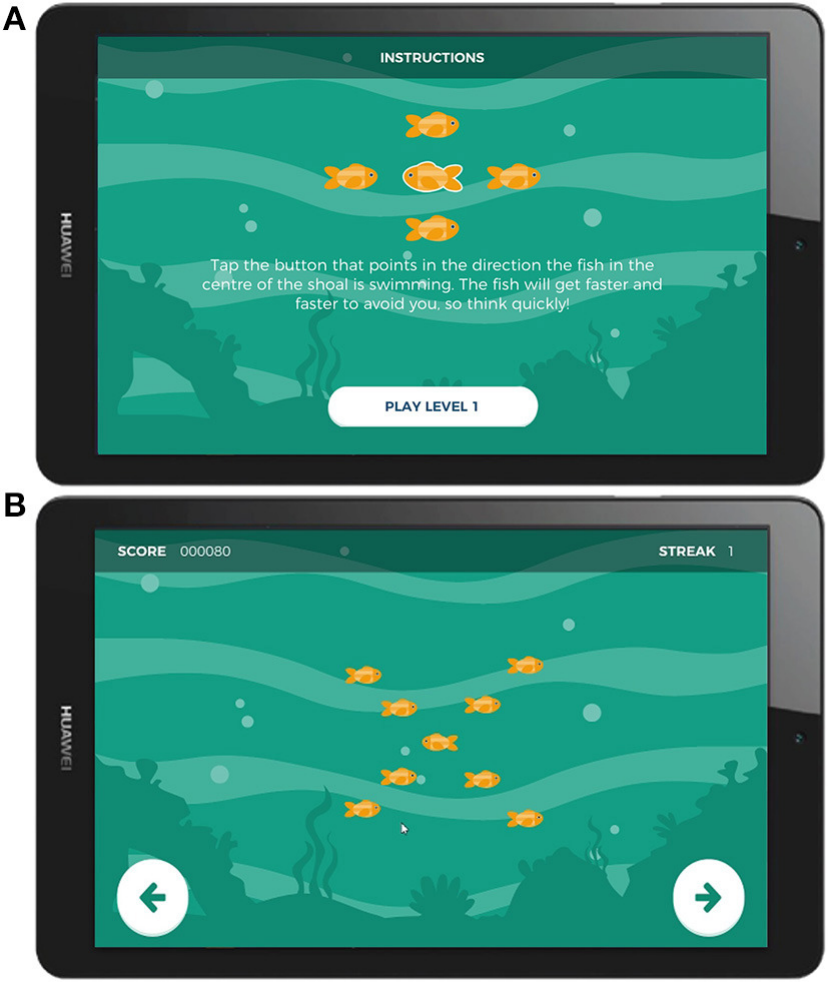
Screenshots of Gamified Adaptive Flanker task. **(A)** Instruction screen, **(B)** Screenshots of the stimulus presentation.

At the beginning of each trial, flanking stimuli—a shoal of orange fish—appeared first on screen, pointing either leftward or rightward, followed 200 ms later by the target (an identical orange fish of the same size) positioned in the center of the array. Participants responded by tapping either the left or right side of the screen, matching the direction in which the central target stimulus was pointing. The flanking stimuli pointed either in the same direction as the target (a “congruent” trial), or the opposite direction (an “incongruent” trial). The background color changed between levels for variety in gameplay. Participants were encouraged not to exclusively prioritize accuracy over speed in two ways: the response time window was shortened with correct responses and lengthened with incorrect responses, and higher points were awarded for quicker responses. These constraints and incentives were designed such that the ideal strategy for maximum points was to respond as quickly as possible and thereby make some mistakes. Gameplay adjustment values were calibrated to produce sufficient numbers of incorrect responses per session (typically between 10–20%) to enable ERP averaging. The task consisted of 150 trials divided into 5 blocks of 30 trials each, evenly split between congruent and incongruent trials. Each session took approximately 7 min to complete. Screenshots of the gamified Flanker task are presented in Figure 2. Task graphics were designed with a color-blind-safe palette.

### EEG acquisition

**Figure 3:**
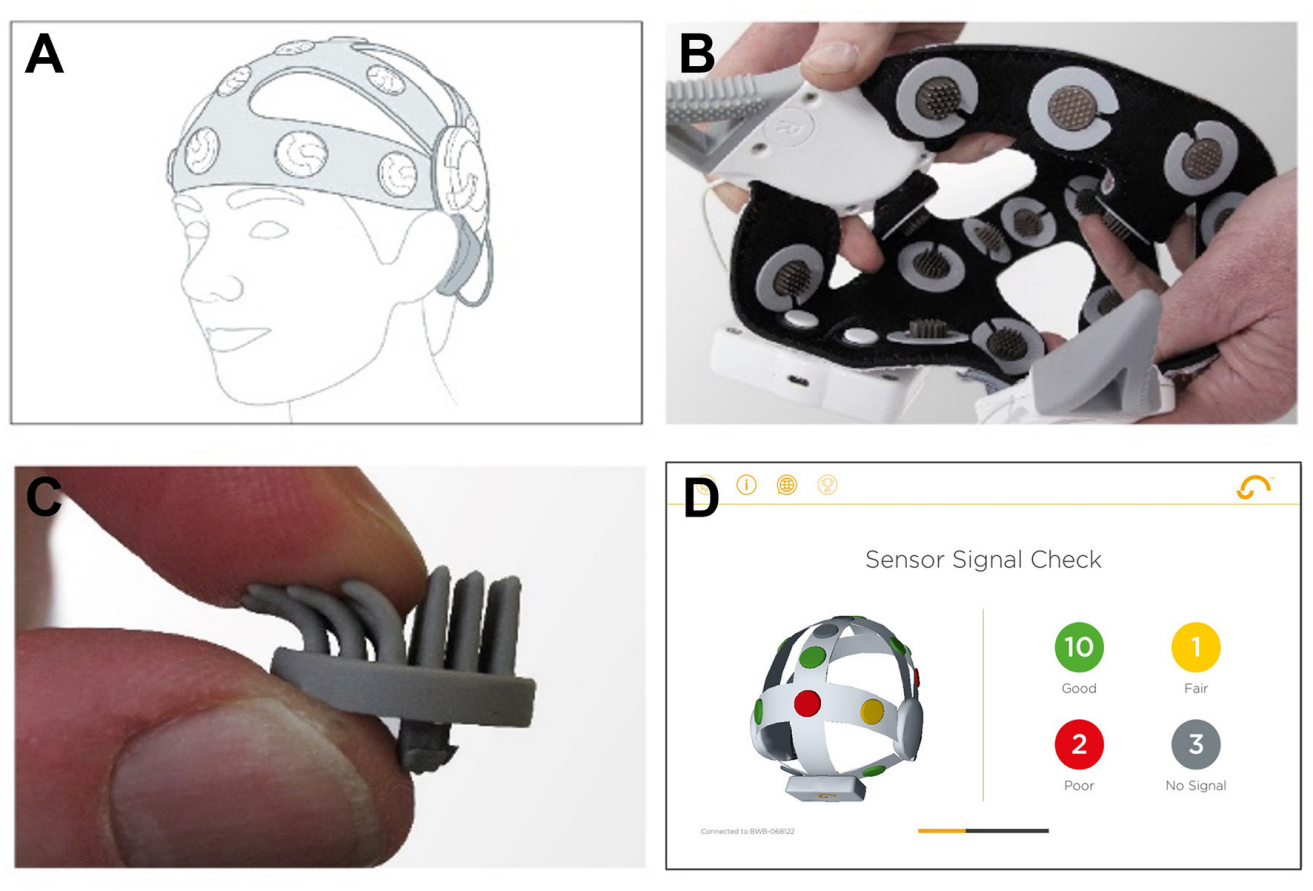
Cumulus dry EEG recording headset. **(A)** Positioning of the headset on the head. **(B)** Interior of the dry EEG cap. **(C)** Dry EEG electrode. **(D)** Screenshot of the built-in calibration step in the mobile app. Figure adapted from McWilliams et al. (52).

We used a wireless 16-channel dry sensor EEG headset developed by Cumulus Neuroscience (Cumulus; www.cumulusneuro.com). Flexible Ag/AgCl coated polymer sensors of a comb-design (ANT-Neuro/eemagine GmbH) were used to achieve a stable and dermatologically safe contact to the scalp at 16 channels (10:10 locations: O1, O2, P3, Pz, P4, Cz, FT7, FC3, FCz, FC4, FT8, Fz, AF7, AF8, FPz; Supplementary Figure S4). The left mastoid was used for reference and the right mastoid for driven-bias, with single-use, snap-on electrodes attached to wires extending from the headset (Figure 3). The headset has an input impedance of 1 GΩ with features including common-mode rejection, and built-in impedance checking. The electronics and sensors are mounted on a flexible neoprene net for comfort and the stretchable structure was designed to enable consistent placement by non-experts in line with the 10-10 sensor system. An onboard processor and Bluetooth module transmitted 250 Hz EEG data to an Android tablet, from where it was transferred to a secure cloud server for storage and processing.

### Procedure

#### Younger adult study protocol

This study was a placebo-controlled, double-blind, randomized, cross-over study designed to investigate the acute and persistent effects of ketamine on EEG and behavioral measures. Participants were invited to the laboratory on five different occasions to complete repeated measurements: at enrolment/screening (visit 1); on the days of infusion of ketamine or saline placebo (visits 2 and 4); and on the days after infusion (visits 3 and 5). The two infusion days took place 4 weeks apart following the same study protocol, while timing of ketamine or saline administration was counter-balanced. Throughout the study, additional task-driven EEG data collection was remotely performed by participants unsupervised in the home, for a week period prior to and after each infusion session (four weeks in total). As the focus of this study was to evaluate usability and fidelity of dry EEG recording, pharmacological effects are not described here: only EEG data collected prior to ketamine administration intervention are analyzed. In contrast, the adherence report encompasses all sessions collected during the entire study.

##### Enrolment session in-lab

The enrolment session, including screening examination, was performed between 21 and 7 days prior to the first infusion session for each participant. After having the purpose and risks of the study explained, all subjects signed an informed consent form, provided a detailed medical history, and completed a screening that consisted of physical, neurological and psychiatric examinations, electrocardiography, and blood draws for clinical laboratory testing (including serology). Then, participants were trained in the use of the recording platform and completed the full suite of tasks plus two additional standard laboratory tasks under dry EEG, all under supervision of a researcher. At the end of the session, participants were given the EEG headset and tablet to take home to carry out their at-home sessions unsupervised. At the enrolment session dry EEG onboarding, familiarization and initial recordings with these two tasks took about 45 min.

##### At-home phase

Participants were instructed to perform daily recordings during the weeks before and after each infusion session, for a total duration of 4 weeks. Pre-infusion recordings served to track initial learning/habituation effects and establish an at-home baseline condition. Post-infusion at-home recordings were intended to explore any prolonged drug effects that persist in the days after infusion. Each recording session (including set-up and calibration for signal quality) lasted approximately 45 min. Participants were instructed to complete their sessions in a quiet place at home around a regular time of the day between 5–9 pm, when they would not be disturbed. The at-home task list included gamified mismatch negativity, visual Oddball, and Flanker tasks, and a 7-min resting state recording with eyes open and closed. Only data extracted from the gamified Flanker and Oddball tasks are presented here.

#### Older adult study protocol

Participants were asked to complete a suite of gamified tasks 5 days per week for 6 weeks: visual Oddball, Flanker, N-back, and delayed match-to-sample tasks, plus resting state, during simultaneous dry EEG recordings, unsupervised in the home using the dry EEG recording platform. Due to the cumulative length of the tasks, they were split across two alternating daily protocols, meaning that the Oddball and Flanker games were scheduled on every second day of participation.

##### Enrolment session in-lab

Participants were invited to the laboratory to be trained on the dry EEG platform use. Upon arrival, they provided informed consent and were fitted to the correct headset size. Participants then completed a training session on how to use the headset and tablet. This training was led by a research assistant and explained how to: log into the application; put on the headset following the in-built, step-by-step instructions of the mobile app delivering the task suite; and a practice run of each game until they felt confident playing the game. After training, all tasks were performed under dry EEG. Participants were then given the EEG headset and tablet to perform the at-home recordings. This initial session lasted ~2 h, with dry EEG onboarding, familiarization and initial recordings with these two tasks took ~1 h. Participants were the sole recipient of the training on how to use the platform (i.e., no study partners or carers were involved in the study nor participated in this onboarding session).

##### At-home phase

Each daily session comprised a resting state recording with eyes open and closed (1 min each) and two of the four games (alternating between sessions). Participants were instructed to complete their EEG sessions in a quiet place at home at any time that would suit them, when they would not be disturbed. Sessions carried in the home lasted <30 min. The gamified Oddball, Flanker and delayed match-to-sample tasks lasted about 12, 7 and 10 min, respectively. Resting state recordings lasted ~2 min, the N-back tasks ~ 5 min. Only data extracted from the gamified Flanker and Oddball tasks are presented here. The order of the gamified tasks were randomized on each day to mitigate order and fatigue effects.

### Mobile app interface

Upon logging into the app, a stepwise tutorial guided participants through the headset configuration (head placement, positioning of detachable mastoid sensors and feedback on electrode impedances) in preparation for recording data during the gamified tasks. Cloud-based secure methods were used for collection and automatic processing of behavioral and EEG data, as well as integration with other data streams (in these studies participants wore a fitness tracker, the Withings Go, https://www.withings.com/) and web-based dashboards for monitoring and data visualization on a daily, session-by-session basis.

### Usability analyses

To quantify the platform usability, we recorded if participants used the hardware correctly when unsupervised, and we measured subjective feedback *via* a usability questionnaire. To assess how often participants were using the recording platform, mean number of at-home sessions completed per week and total mean numbers of sessions per participant were computed. To assess participants' ability to use the recording platform unsupervised, we computed the percentage of sessions that had successfully been conducted in the home. Success was defined as complete EEG and behavioral log files containing all the tasks of the day. We report the mean percentage of successful sessions per participant. Participants' subjective feedback on the technology was captured *via* the *System Usability Scale*, a 10-item industry standard questionnaire (58) designed specifically to develop and assess the use of technology in industry. In both studies, the completion of the SUS questionnaire was optional and performed at the end of the study after the participants returned their hardware. It was administrated by the researchers either over the phone when participants mailed back their equipment, or in-person when participants dropped by the laboratories to return their headset and tablet. SUS scores from the younger and older adults were compared using one-sided non-parametric Mann-Whitney U rank test to evaluate whether age could modulate usability.

### EEG analyses

The total number of sessions analyzed and contributed by each cohort is described in Supplementary Table S1. Analyses conducted on the Younger Adult Study dataset only included data collected during the week before the pharmacological interventions to avoid any confounding effects.

#### EEG preprocessing

At the end of each session, the EEG data were automatically uploaded to the cloud and the proprietary processing pipeline developed by the technology provider was applied. This was designed to verify and correct the integrity of timing information and exclude bad quality signal portions. Corrective procedures were applied for missing and anomalous data, including eye and other characteristic artifacts. After that, EEG signals were pre-processed with filtering from 0.25–40 Hz, epoch extraction, and baseline adjustment. All data were recorded with a left-mastoid reference. Oddball task epochs were extracted from 500 ms before target stimulus presentation to 1,000 ms after. ERP epochs were baseline adjusted at each electrode by subtracting the mean signal in the 100 ms time window preceding stimulus presentation. Epochs from the Flanker task were extracted from 500 ms before participant's responses to 1,000 ms after. ERP epochs were baseline adjusted at each electrode by subtracting the mean signal in the 100 ms time window from 500 to 400 ms before a participant's response.

As an additional step to improve data quality, noisy epochs were then identified and removed. Epochs were selected per channel following a stepwise procedure. First, epochs with an absolute voltage >100 μV were rejected. In each remaining epoch, ERP amplitude was correlated with the session average (the data were ERP amplitudes at each timepoint). Any epoch with a correlation to the session average of <0.25 was rejected. This threshold was selected to allow for variability in the retained data while rejecting trials that diverged the most from the session average. Following this, a z-score-based data-cleaning approach was used to further remove outliers. To do this, a set of temporal and spectral metrics were calculated and z-scores for each metric were obtained. These temporal and spectral metrics consisted of the Hurst exponent, kurtosis, median gradient value, range, variance, standard deviation, and ratio of high frequency to low frequency power. These metrics are commonly used in EEG data selection steps (59). First, all epochs with z-scores >15 in any metric were rejected. Next, any epochs with a Hurst exponent <0.65 or the spectral peak <0.1, respectively, were rejected. Finally, any remaining epochs with z-score >4 in any metric were rejected. Median ERP amplitudes were then calculated as it is a robust measure of central tendency.

#### EEG data collection success rate computation

Dry EEG signal quality depends greatly on the specific hardware and how it is used. To check the amount of usable data that was kept after the preprocessing and trial selection steps described above, we calculated success rates of data collection. We report the mean number of trials available per participants for each task and condition. The number of sessions surviving after the preprocessing and trial selection steps (as all trials in a session could be rejected) were used to calculate the success rates, as a percentage of the total number of sessions recorded. For each task, the percentage of successful sessions was computed for all participants in both studies.

#### EEG signal variability quantification

##### At-home data variability

To evaluate signal quality as a function of the number of trials available, we computed the *SME*_*i*_ for each participant *i* and for various number of trials according to the following equation (60, 61):


(1) $$\begin{array}{r} SME_{i}=\;\frac{\sigma_{i}}{\sqrt{n_{i}}} \end{array}$$

where σ_*i*_ corresponds to the standard deviation of the metric of interest, and *n*_*i*_, to the number of trials available. Luck and colleagues define the SME as “*the standard error of measurement for an ERP amplitude or latency score.”* To keep our analyses centered on data quality and avoid having to account for the variability introduced by different peak selection approaches, we focused on the time-window mean amplitude approach (60). ERP amplitudes extracted from the Oddball task were calculated by taking the mean voltage amplitude across the 300-400 and 400-500 ms windows after stimulus presentation for Target trials for the Younger and Older Adult study, respectively (see **Figures 7**, **8** for the suitability of these parameters; the older adult P300 peaked ~100 ms later than the younger adult P300). Analyses of the Oddball and Flanker tasks were focused on electrodes Pz and FCz, respectively. These electrodes were chosen as the P300 and ERN signals are expected to be maximal around central and fronto-central regions, respectively (62). ERP amplitudes extracted from the Flanker task were calculated by taking the mean voltage amplitude between 0–100 ms time windows after participants' response for both Correct and Incorrect trials. The standard deviation of these time-window mean scores σ_*i*_ was then used to compute the *SME*_*i*_. The trials used for the calculation were added in sequential order, meaning that trials were aggregated as they were recorded: when calculating the SME for *n* trials, the first *n* available trials of a participant were used.

We report group-level SME for increasing numbers of at-home trials, aggregated over multiple sessions. The corresponding 95% confidence intervals were computed as the standard error of the SME across all participants, divided by the square root of *N*, the number of participants. We note that as we increased the number of trials to aggregate, fewer participants had sufficient data to be included and we quantify this in each analysis. SME is compared to the value achieved in the wet EEG reference dataset, and the number of trials in wet and dry studies, after their respective trial rejection procedure.

Finally, we tried to answer the question of how much at-home gamified dry-EEG data is as good or better than a single session's worth of laboratory-derived wet-EEG data. This is not straightforward, as the duration of tasks and rate of trial presentation were not the same in wet and dry settings. However, we did project how many pre-rejection trials of home dry-EEG data would be required to achieve an SME level similar to laboratory wet-EEG, and therefore how many additional sessions of dry EEG data collection would be required.

##### Data quality of dry EEG recordings collected under supervision in the laboratory

To evaluate the impact on data quality of completing the dry EEG recordings in the laboratory in a controlled environment with the supervision of a trained technician, we computed group-level SME for sessions completed in the laboratory for both cohorts. The older cohort only completed one session in the laboratory during their initial on-boarding study visit. For the younger cohort, 3 sessions were completed in the laboratory prior any drug administration: during the initial on-boarding study visit and before the infusion of the ketamine and placebo solutions. We report group-level SME for increasing numbers of in-laboratory trials. The corresponding 95% confidence intervals were computed as the standard error of the SME across all participants, divided by the square root of *N*, the number of participants.

### Wet EEG dataset

Wet EEG reference SME values were extracted from the open-source ERP CORE EEG database provided by Kappenman et al. (62) using Equation (1). The objective of this study was to provide the community with an optimized reference dataset performed by experts in the field of ERP experimentation. Processed and raw data as well as the experiment control scripts can be downloaded at https://doi.org/10.18115/D5JW4R and the details of their Flanker and Oddball tasks have been described in a previous publication (62). In brief, EEG data was collected from 40 participants (25 females, mean age: 21.5 years old) who completed six 10-min optimized paradigms including an Oddball and a Flanker task. In the active visual oddball task, 4 letters (A, B, C, D, E) were presented in random order and for each block, with one of the letters designated as the Target, and presented with a probability of 0.2. In the Flanker task, a central arrowhead was the target stimulus and was flanked on each side by a set of arrowheads. These flanking stimuli pointed either in the same direction as the target (a congruent trial) or the opposite direction (an incongruent trial). A Biosemi ActiveTwo recording system with 128 active electrodes (Biosemi B.V., Amsterdam, the Netherlands) was used to collect the data. An average of electrodes P9 and P10 (located near the mastoid) was used to reference the data. Semi-automatic algorithms were used to remove large muscle artifacts, extreme voltage offsets, or break periods longer than 2 s. After that, Independent Component analysis (ICA) was performed, and components associated with eye movements (including eye blinks) were removed. The remaining components were then remixed and projected back to electrode space. These trials were further examined using individualized thresholds and any trial still containing large voltage excursions and large eye movements were excluded. SME values were computed at electrode Pz and across the 300–600 ms time window post stimulus presentation for the Oddball task, and at FCz across the 0–100 ms time-window post participants' response for the Flanker task. SME data, derived from the open-source data, were provided by Prof. Steven Luck's group (personal correspondence 02/04/2022).

## Results

### Participants

Of 56 participants recruited in the Older Adult study, 50 commenced and completed the study (see Table 1 for details). Six participants were excluded for the following reasons: two did not meet the inclusion criteria (one was mistakenly enrolled, and another revealed taking medications after enrolment), three were ill during the at-home data collection (unrelated to this study), and one person could not be accommodated due to head size.

**Table 1:** Participant demographics.

	**Gender**	**Mean age in years** **(SD, range)**	**Mean MoCA score** **(SD)**	**Mean years of education** **(SD)**
Older adult study	25 males 25 females	67.84 (5.03, 58–81)	27.22 (2.04)	15.24 (3.6)
Younger adult study	30 males	25.56 (3.74, 18–36)	Not collected	Not collected^a^
Wet EEG study (65)	25 females 15 males	21.5 (2.87, 18–30)	Not collected	Not collected

SD, standard deviation.

a 83% of the sample were current students.

In the Younger Adult study, 36 participants were initially recruited but two were excluded for medical reasons and four withdrew. The reasons given for withdrawal were the following: one person expressed discomfort while wearing the headset, one expressed anxiety about the blood draws, one expressed anxiety about taking the drug, and one felt sick during the study (see Table 1).

### Usability

**Figure 4:**
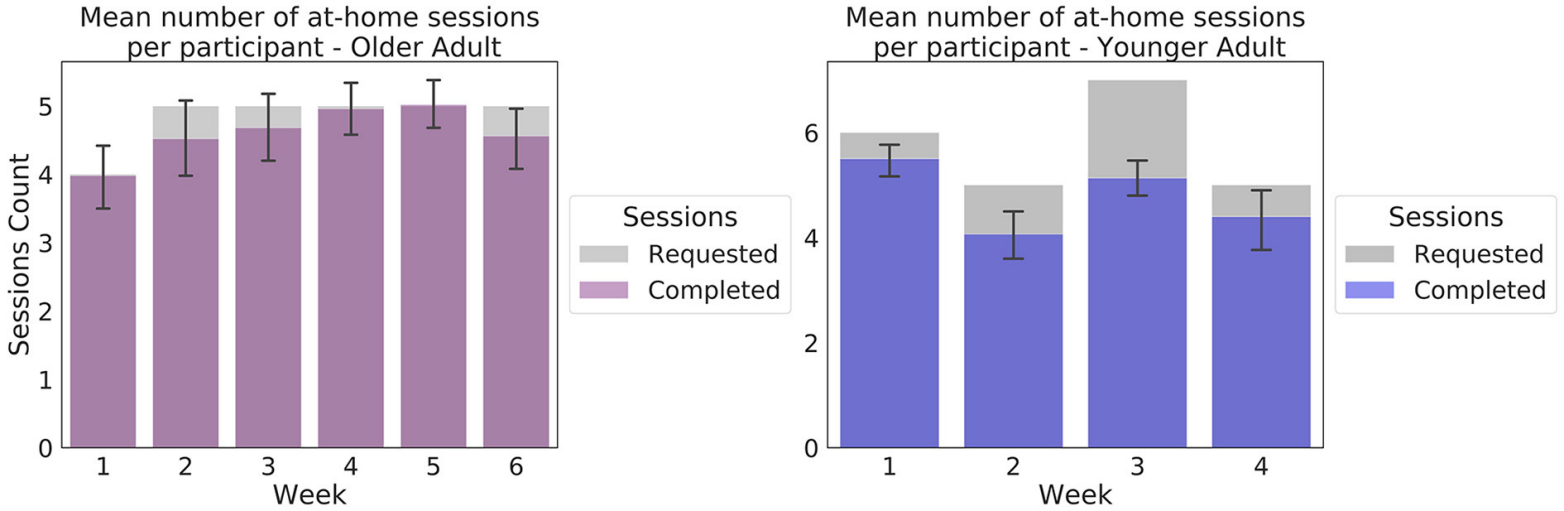
Weekly at-home adherence to the experimental protocol. Data in purple correspond to the Older Adult study (*N* = 50). Data presented in blue correspond to the Younger Adult study (*N* = 30). Error bars represent 95% confidence intervals. The gray areas correspond to the number of at-home sessions requested in the protocol. The number of sessions requested varied as per protocol, to accommodate scheduled in-lab sessions.

In the Younger Adult study, 965 sessions were collected, including 281 sessions collected in the laboratory around the enrolment and infusion sessions. On average, participants attempted 22.87 sessions (SD = 1.92, range: 16–24), of 23 at-home sessions requested (including data from 17 participants who submitted extra sessions). Of 30 participants, 22 attempted the 23 sessions requested. On average, per participant, 96% of the sessions (SD = 5%, range: 79–100%) resulted in a complete EEG/behavioral dataset for input into the preprocessing pipeline. Of 30 participants, 10 successfully completed 100% of their attempted sessions. Weekly adherence results are presented on Figure 4.

In total, 1,499 EEG sessions were collected in the Older Adult study, of which 50 were collected in the laboratory during the enrolment sessions. On average, participants attempted 28.9 at-home sessions (SD = 6.64, range: 10–49), of 29 at-home sessions requested (including sessions from the 24 individuals who submitted more sessions than were requested). Of 50 participants, 33 participants attempted the 29 sessions requested. On average, per participant, 93% of sessions commenced (SD = 7%, range: 73%-100%), resulted in a complete EEG/behavioral dataset for input into the preprocessing pipeline. Of the 50 participants, 12 successfully completed 100% of their attempted sessions.

**Figure 5:**
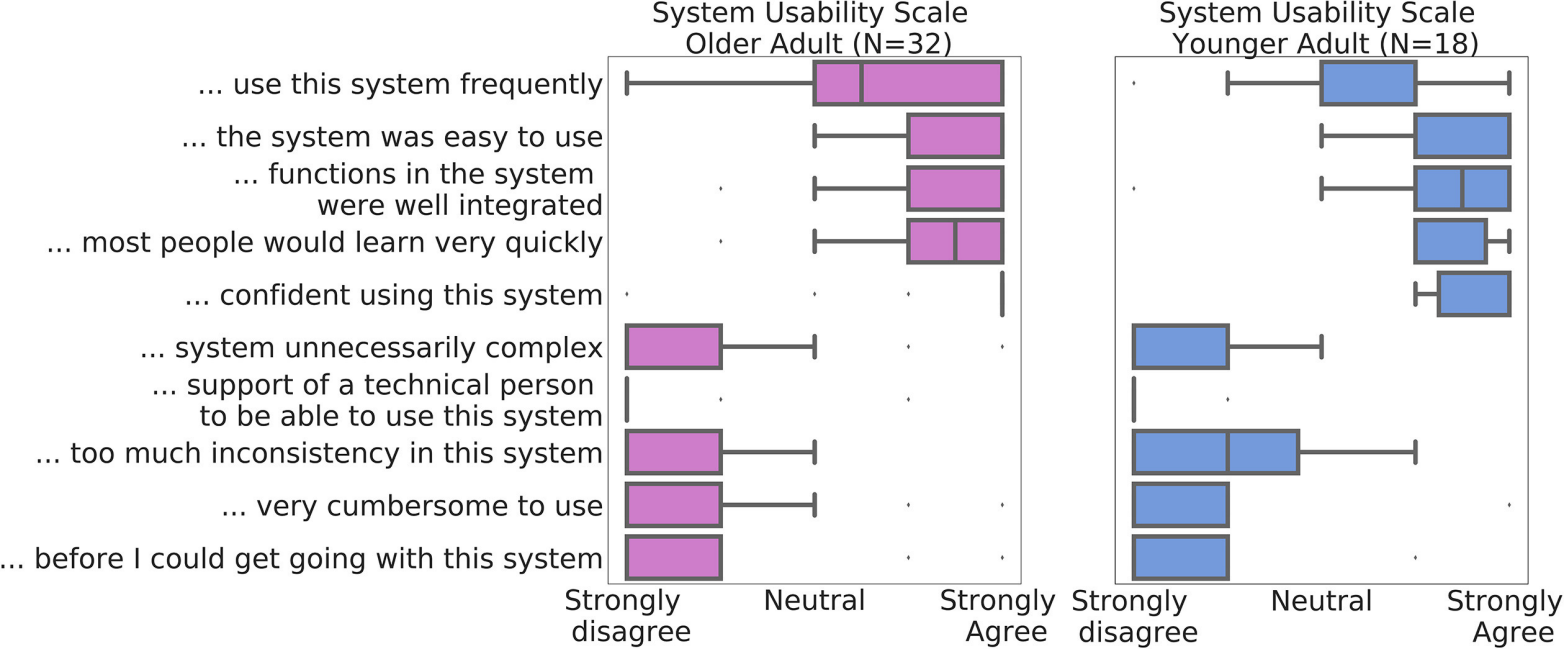
Median System Usability Scale component scores across all participants in the Data of Older Adult study are depicted in purple (*N* = 32), and of the Younger Adult Study in blue (*N* = 18). Whiskers denote interquartile range. Datapoints outside of the 1.5*interquartile ranges were defined as outliers.

At conclusion of the studies, 32 of the older participants and 18 of the younger participants chose to complete the optional debrief session, including the self-report SUS usability scale. The mean SUS score at the end of the Older Adult study was 84.53 (SD = 10.15) and 88.33 (SD = 10.18) at the end of the Younger Adult study (Figure 5). The individual response components of the scale are displayed in Figure 5. No significant differences were observed between the younger and older cohorts in the main SUS composite score, or any of the individual components (full results are presented in Supplementary Table S2).

### EEG Results

#### EEG data collection success rate

Table 2 summarizes the number of trials collected for Oddball and Flanker tasks in the two studies and the wet EEG dataset, and how many of those trials survived epoch rejection to reach analysis at key electrode locations.

**Table 2:** Mean number of available trials after processing and percentage of data discarded per session per participant.

			**Number of trials collected**	**Percentage of trial discarded (%)**	**Number of available trials after artifact rejections**
			**(SD, range)**	**(SD, range)**	**(SD, range)**
*Older adult study* * (N = 50)*	Oddball task	Target trials	30	64.2 (26.5, 0–100)	10.7 (7.9, 0–30)
	Flanker task	Correct trials^a^	126.2 (4.8, 64–134)	73.9 (21.8, 6.5–100)	32.6 (27.4, 0–117)
		Error trials^a^	6.7 (4.1, 1–24)	61.9 (36.4, 0–100)	2.5 (2.8, 0–16)
*Younger adult study* *(N = 30)*	Oddball Task	Target trials	30	43 (23.4,0–100)	17.1 (7, 0–30)
	Flanker task	Correct trials^a^	117.8 (9.6, 85–136)	59.3 (18.7, 4.7–100)	47.5 (21.9, 0–104)
		Error trials^a^	20.2 (13.3, 1–64)	40 (24.7, 0–100)	11.3 (8.2, 0–39)
*Wet EEG dataset* *[N = 40; Kappenman et al. (62)]*	Oddball task	Target trials	40	23.69 (21.2, 0–87.5)	30.5 (8.5, 5–40)
	Flanker task	Correct trials^b^	352.1 (36.2, 182–398)	4.5 (7.3, 0–34.9)	337 (46.2, 151–390)
		Error trials^b^	42 (22.4, 2–83)	4.3 (7.2, 0–33.3)	40.1 (21.4, 2–80)

The number of trials passing quality control were computed at the Pz electrode for the Oddball task, and at the FCz electrode for the Flanker task. The wet EEG tasks lasted about 10 min. The gamified Oddball and Flanker lasted about 12 and 7 min, respectively.

a 150 trials were presented and could be answered correctly or incorrectly.

b 400 trials were presented and could be answered correctly or incorrectly.

**Figure 6:**
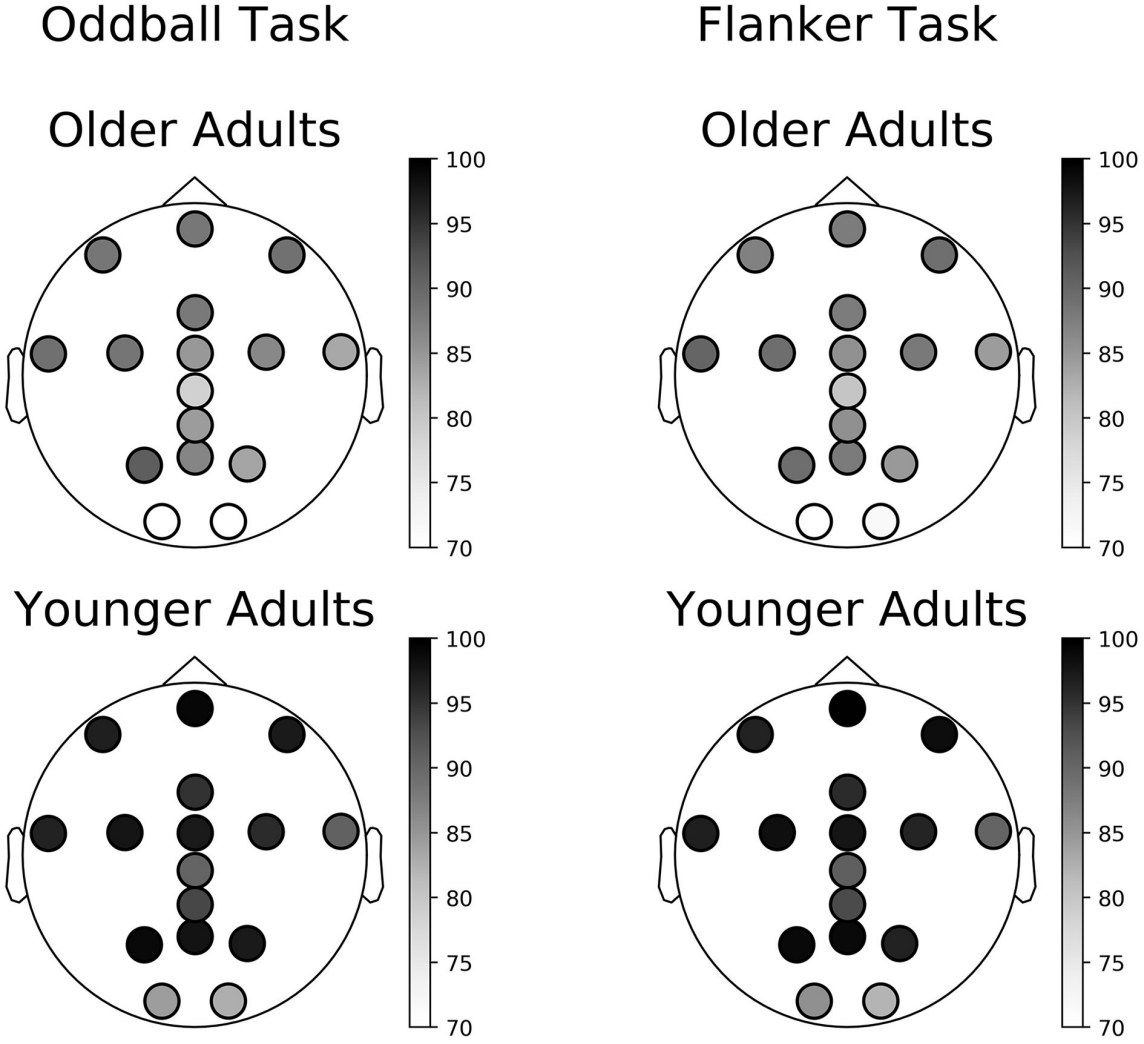
Percentage of available sessions after pre-processing per channel. The top row corresponds to data collected in the Older Adult study. The bottom row corresponds to data collected during the Younger Adult study. The left column of each figure corresponds to data collected during the gamified Oddball task, the right column of each quadrant to data collected during the gamified Flanker task.

In some cases, all trials were rejected, and a session was not available for analysis. Of tasks for which the data was complete in the Younger Adult study, on average across all electrodes, 95% of the Oddball task sessions (SD = 5%, range: 82–100%) and 94% of the Flanker task sessions (SD = 6%, range: 83–99%) remained following pre-processing (results are presented on Figure 6 and Supplementary Table S1). In the Older Adult study, on average across all electrodes, 84% of completed Oddball task sessions (SD = 7%, range: 68–91%), and 85% of the Flanker task sessions (SD = 6%, range: 69–90%) were retained following pre-processing (see 2.8.1; results are presented on Figure 6 and Supplementary Table S1).

To check that the number of trials rejected remained stable across sessions, we plotted the number of trials rejected across sessions at FCz and Pz in both cohorts. Percentages of trials rejected across sessions are presented on Supplementary Figure S3. A summary of the number and percentage of available sessions at each step of the data analyzes are presented in Table 3.

**Table 3:** Number of available at-home sessions at each step of the data collection analysis, including percentage retained relative to previous step.

**At-home sessions**	**Older adults**			**Younger adults** ^a^		
	**Session**	**%**	**Cumulative %**	**Session**	**%**	**Cumulative %**
**Oddball task**						
Requested	725	100	100	390	100	100
Attempted	750	103.4	103.4	347	89.0	89.0
Task data complete	730	97.3	100.7	346	99.7	88.7
Survived epoch rejection^b^	634	86.8	87.4	339	98.0	86.9
**Flanker task**						
Requested	725	100	100	390	100	100
Attempted	699	96.4	96.4	347	89.0	89.0
Task data complete	675	96.6	93.1	347	100.0	89.0
Survived epoch rejection^b^	577	85.5	79.6	339	97.7	86.9

The number of sessions that passed quality control were computed at the Pz electrode for the Oddball task, and at the FCz electrode for the Flanker task.

a Only data collected before the infusion interventions are evaluated here, therefore percentages may vary somewhat relative to the figures cited in section Usability.

b All the epochs in a session could be rejected during this step.

#### Younger adult study

**Figure 7:**
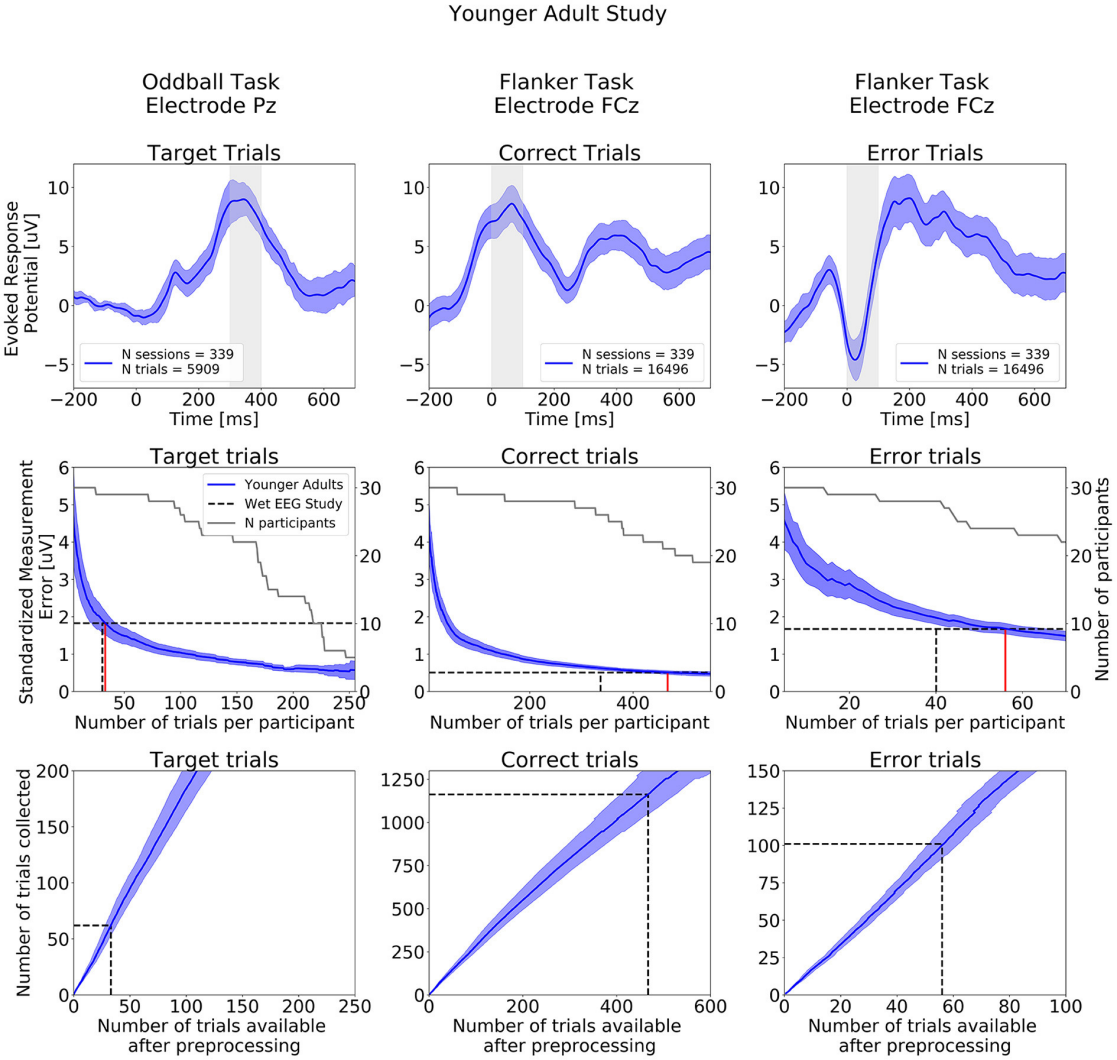
ERPs analyses extracted from the Younger Adult Study (*N* = 30). Top row: Grand study average computed across all participants. Middle row: Standardized Measurement Errors per number of trials. The gray continuous lines correspond to the number of participants remaining in the SME calculation after trial rejection. Bottom Row: Mean numbers of trials available after preprocessing. The black dotted lines correspond to the mean numbers of trials extracted from the wet EEG study. From left to right: Target Trials extracted from the Oddball task at electrode Pz, Correct Trials extracted from the Flanker task at electrode FCz, Error Trials extracted from the Flanker task at electrode FCz. The shaded areas correspond to the 95% confidence intervals.

ERPs extracted from the Oddball and Flanker task of the Younger Adult Study are presented in the top row of Figure 7. ERPs extracted at all electrodes are presented on Supplementary Figures S5–S7. The typical waveform features of a P300 ERP can be observed: negative readiness potentials, P2 and N2 components occurred from before 0 to 250 ms, followed by the P300 component peaking around 350 ms. When examining data from the Flanker task we observe the expected positive deflection after Correct Trials and negative deflection after Error trials around 50 ms after participants' response.

The central row of Figure 7 projects what SME can be achieved by aggregating increasing numbers of trials per participant over multiple at-home dry sessions. As noted above, as that number increases, the number of participants that can be included in the sub-analysis decreases (as some participants may not have collected enough data to be included in the sub-analyses). The SME can be seen to decrease with the inverse of the root mean square of numbers of trials.

The wet EEG study (62) reported achieving an SME of 1.83 μV based on the average of 30.5 Oddball target trials that survived their epoch rejection strategy (gray-dotted lines in the figure). Comparing that to data from our Younger adult at-home data, we found that 33 Target trials (red line in the figure) were required in the Younger Adult Study an equivalent level of ERP variability (though with the higher rate of epoch rejection previously mentioned).

For the Flanker task, the wet EEG study reported achieving SME values of 0.51 μV and 1.68 μV for Correct and Error trials, respectively. We found that 467 Correct trials and 56 Error trials were required to reach the same SME values obtained with 337 Correct trials and 40 Error trials using a wet EEG system (all following epoch rejection).

The bottom row of Figure 7 shows the total number of experimental trials required to reach wet-EEG benchmark SME values for each ERP, and we can project from that to the number of at-home sessions to achieve or exceed that (based on trial numbers and rates of rejection in Table 2). For this study, which had a comparable cohort to the wet EEG dataset, we can project that 2 equivalent sessions of at-home gamified dry EEG would provide a superior SME (lower signal variability) than the single lab-based session for the Oddball Target—assuming the number of trials per session were the same in both studies. For the Flanker task 3 dry EEG sessions would be sufficient for the Error trials ERP, and 4 sessions for the Correct trial ERP.

**Figure 8:**
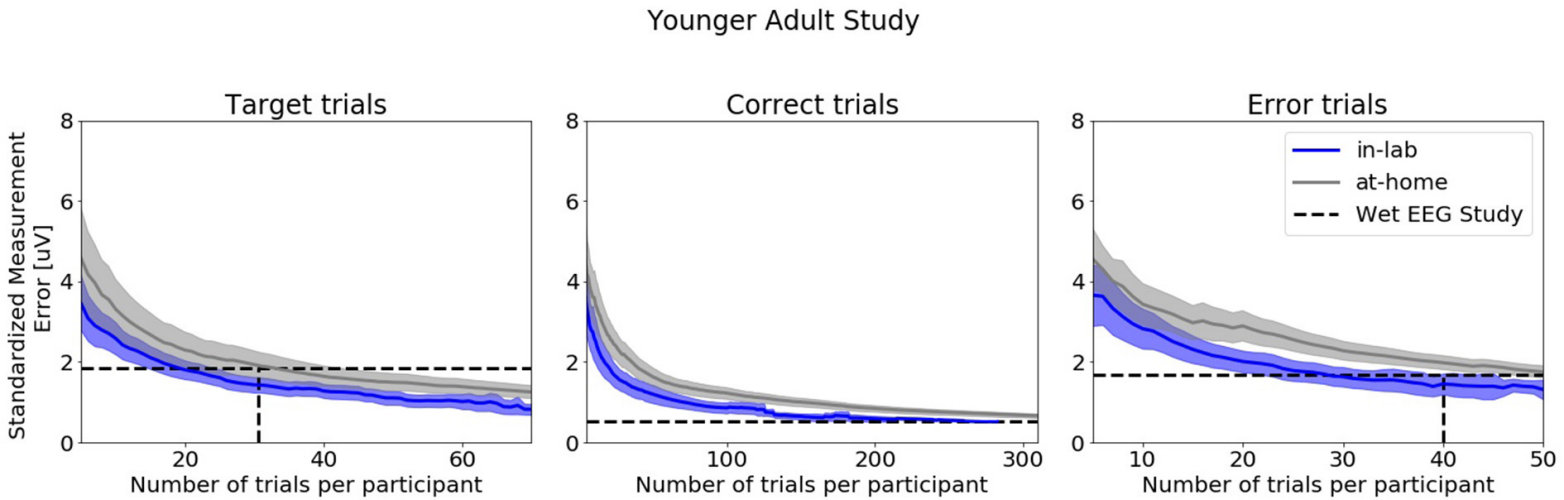
Standardized Measurement Errors per number of trials of data collected in the laboratory under researcher supervision extracted from the Younger Adult Study (*N* = 30). The blue line corresponds to data collected in the laboratory. The gray line corresponds to data collected in the home. The black dotted lines correspond to the mean numbers of trials extracted from the wet EEG study. From left to right: Target Trials extracted from the Oddball task at electrode Pz, Correct Trials extracted from the Flanker task at electrode FCz, Error Trials extracted from the Flanker task at electrode FCz. The shaded areas correspond to the 95% confidence intervals.

Figure 8 shows SME data from the in-laboratory sessions for both the Oddball and Flanker tasks.

#### Older adult study

**Figure 9:**
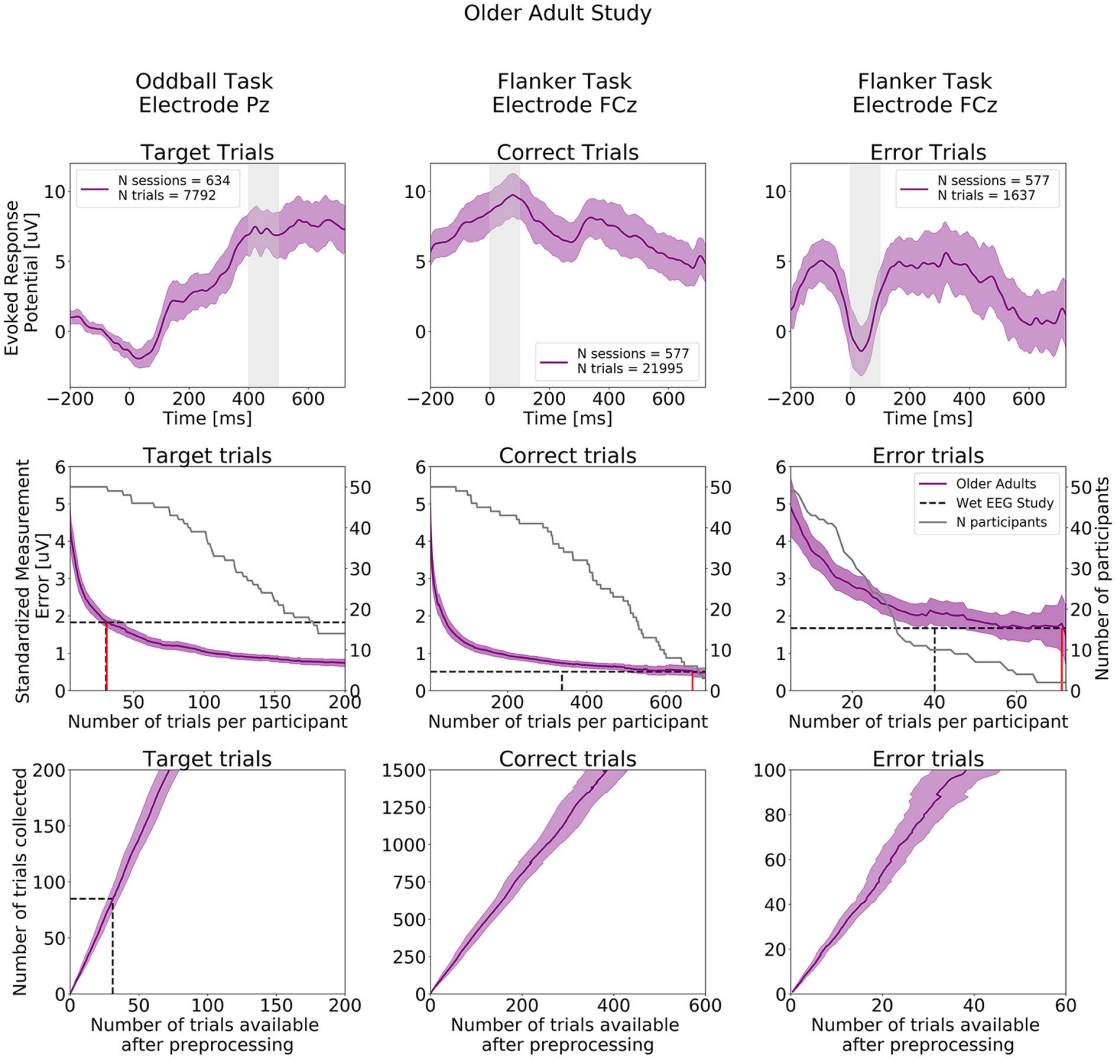
ERPs analyses extracted from the Older Adult Study (*N* = 50). Top row: Grand study average computed across all participants. Middle row: Standardized Measurement Errors per number of trials. The gray continuous lines correspond to the number of participants remaining in the SME calculation after trial rejection. Bottom Row: Mean numbers of trials available after preprocessing. The black dotted lines correspond to the mean numbers of trials extracted from the wet EEG study. From left to right: Target Trials extracted from the Oddball task at electrode Pz, Correct Trials extracted from the Flanker task at electrode FCz, Error Trials extracted from the Flanker task at electrode FCz. The shaded areas correspond to the 95% confidence intervals.

ERPs extracted from the Oddball and Flanker tasks from the Older Adult Study are presented in the top row of Figure 9 and ERPs at all electrodes are presented in Supplementary Figures S8–S10. As in the Younger Adult Study, Negative readiness potentials, P2 and N2 components occurred from before 0 to 250 ms, followed by the P300 component peaking a bit later compared to the younger adult at about 450 ms in the ERPs extracted from the Oddball task. As before, we observe in the Flanker task data, the expected positive deflection after Correct Trials and negative deflection after Error trials around 50 ms after participants' response.

The central row of Figure 9 projects the SME that can be achieved in the Older Adult Study. When comparing with SME values extracted from the wet EEG study, 31 Target trials were required in the Older Adult Study to reach the same SME value obtained with 30.5 Target trials using the wet EEG system. For the Flanker task, 667 Correct trials and 71 Error trials were required to reach the same SME values obtained with 337 Correct trials and 40 Error trials in the wet EEG study.

The bottom row of Figure 9 shows the total number of experimental trials from our older cohort required to reach wet-EEG SME values from a younger cohort. Here, 3 dry EEG sessions for the Oddball Target, and 7 at-home sessions are sufficient for both Error and Correct ERPs in the Flanker task, would have provided a superior SME (lower signal variability) than the single lab-based session if the same number of trials were collected per session with the wet and dry EEG system.

**Figure 10:**
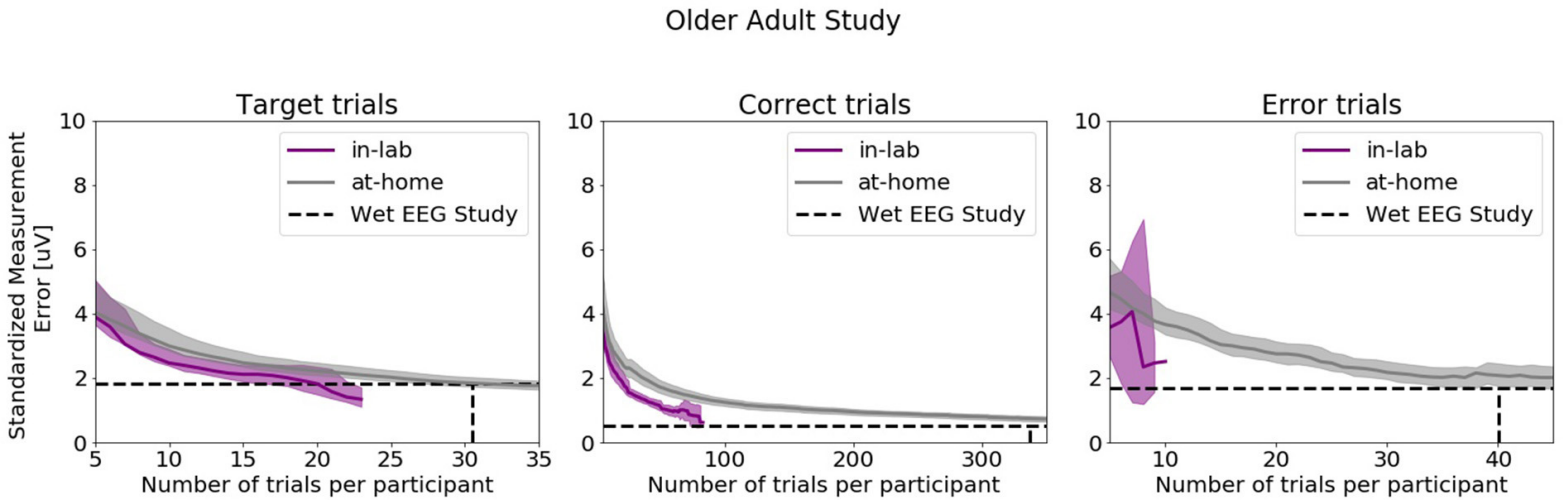
Standardized Measurement Errors per number of trials of data collected in the laboratory under researcher supervision extracted from the Older Adult Study (*N* = 50). The purple line corresponds to data collected in the laboratory. The gray line corresponds to data collected in the home. The black dotted lines correspond to the mean numbers of trials extracted from the wet EEG study. From left to right: Target Trials extracted from the Oddball task at electrode Pz, Correct Trials extracted from the Flanker task at electrode FCz, Error Trials extracted from the Flanker task at electrode FCz. The shaded areas correspond to the 95% confidence intervals.

Figure 10 shows SME data from the in-laboratory sessions for both the Oddball and Flanker tasks.

## Discussion

The overall objective of this study was to evaluate if dry EEG recorded in the home by unsupervised participants, with easy-to-use engaging tasks, could yield datasets of the same quality as published results derived from wet EEG recorded by an expert group in a controlled environment. The primary finding was that it was feasible to reach similar SMEs compared to a wet EEG study with the same numbers of dry trials when comparing populations of similar age. However, the percentage of trials discarded after artifact rejection was substantially higher in the dry system compared to what was reported in the wet EEG study used as reference. We also evaluated the recording platform usability using the participants' adherence to the protocol, successful data collection rate, and subjective reports. Participants reliably collected EEG data on a near-daily basis.

Participants exhibited high adherence to the dry EEG protocol: on average >94% of requested sessions were commenced in both cohorts. Notably, the compensation of each cohort was not pro-rated to the number of sessions session completed in the home. The older group were modestly compensated (€40) for the at-home study, whereas the younger group were paid €500 for their participation in the study: suggesting that monetary compensation alone was not the most important factor in adherence. There was a high rate (over 93%) of sessions successfully completed at home. Furthermore, the positive user feedback [mean SUS score >84 in the older cohort and >88 in the younger cohort; corresponding to “good/excellent” ratings (58)] demonstrated that participants, including older adults up to 81 years-old, did not appear to feel the lack of supervision of a trained technician to complete neurocognitive tasks. No clear evidence of usability barrier in the older cohort was found as attested by the lack of differences in usability scores between the two groups. This result is in agreement with findings from Nicosia and colleagues who reported that, while older age is associated with less technology familiarity, older adults are willing and able to participate in technology-enabled studies (32). The high percentage of successful sessions in the older cohort also underlines that the ease-of-use of the platform was satisfactory and enabled populations who were not digital natives to use the system. Notably, there was no substantial drop in adherence during later phases of the studies. These results are consistent with previous findings from McWilliams and colleagues who deployed the same dry EEG technology in a separate cohort of 89 healthy older adults and reported high adherence and usability scores (e.g., mean adherence of 82% and mean SUS score of 78.7) (52). Our compliance levels are also slightly higher than those observed in purely cognitive remote studies using smartphone for repeated testing [e.g., adherence of 85.7% (32)]. As discussed by Moore et al., it is likely that our older participants' high adherence rate is due to a combination of extrinsic factors including the ease-of-use of the technology, and intrinsic factors such as personal motivation (the older cohort self-referred into our study) (63). No carer or study partner attended the initial in laboratory on-boarding visit suggesting that a single in-person training session was sufficient for older adults to commence at home recording. However, we do not know if participants were helped by another person once in the home.

In the context of neurodegenerative research, the dry EEG approach described here could be especially useful for prodromal phase studies as change can emerge over years (64). One could imagine asking an at-risk population to complete 2 weeks of recordings every year to detect subtle changes in brain function and cognition. The P300 latency has, for example, been proposed as a marker of cognitive decline in older population and appears to be sensitive to disease stage within the context of Alzheimer's type of dementia (14, 65, 66). The observation of the classical features of the ERN and P300 in both cohorts suggest that gamified dry EEG can capture these ERP components. These results taken together suggest that intermittent at-home EEG recording is well tolerated by older adults and would make the monitoring of such markers longitudinally possible.

The comparison of the ERP CORE wet EEG data evaluated participants of similar age profile to our younger adult cohort revealed that similar numbers of trials were required to reach the same SME values. However, on average (and as expected), more dry EEG trials were rejected during preprocessing (between 2 to 14 times more). This result demonstrates that applying stringent data rejection techniques to dry EEG data makes it possible to generate averaged ERPs trials similar to those collected in laboratory environments. When accounting for the number of trials lost during artifact rejection, our analyses suggest that between 2 to 4 at-home gamified dry EEG sessions using the same tasks parameters would yield the same SMEs as the wet EEG system for comparable cohorts. The main factor guiding the decision of how many trials should be collected in traditional in-clinic wet EEG studies is the quality and variability of the signal. The burden of the tasks is a secondary concern. Indeed, as participants are in the laboratory once or twice only and as the hardware can be cumbersome to configure, researchers aim to collect as many trials as possible in the shortest amount of time. When designing tasks for repeated self-administered measurements in the home, the recordings need to be short and enjoyable enough so that participants are inclined to conduct additional sessions (26). Thus, considering this factor combined with the fact that dry EEG data are inherently noisier than wet data because of the dry electrode higher impedance, it is unsurprising to find that one must aggregate over a number of sessions to reach equivalent quality levels. These observations are also consistent with behavioral findings from studies evaluating online platforms and mobile-apps (39, 67–71). For example, Lipsmeier and colleagues were able to distinguish healthy controls from individuals with Parkinson's disease by aggregating sensor-based features collected from mobile phones over 14 days (71).

The percentage of data that survived the different data selection procedures varied as a function of cohorts, tasks, and electrode location. Overall, dry EEG data collected from older adults were noisier than data from younger adults: between 15 to 20% more trials were rejected during the data selection procedures. Further investigations are needed to disentangle the origin of these differences, but one working hypothesis is that it could be due to age-related impairment in manual dexterity (72) and its effect on headset setup and sensor contact quality. When comparing Flanker data extracted from the Younger and Older Adult studies, we observed that on average, each older adult session yielded less data compared to the young one. Two factors seem to be driving this effect, on average: i) more data were rejected from the Older Adult study and ii) older adults committed 3 to 4 times fewer errors compared to younger adults. The latter result suggests that older adults may use different speed vs. accuracy trade-off strategies compared to younger adults (73) and this should be taken into consideration when planning for the number of trials to be collected in studies within older populations.

Finally, we also discovered that the yield of usable data varied as a function of the electrode location and that Cz, O1, and O2 yielded the lowest amounts of usable data, possibly due to individual variation in head shape and hair style. These results suggest that future headset design should consider potential solutions to this problem such as adjusting the length of the pins, changing the morphology of the electrodes at these locations, or making the headset more adjustable.

In the context of drug clinical trials, recording across a week prior to a therapeutic intervention could establish a baseline of brain activity more robust to daily fluctuations (e.g., caused by lifestyle factors). Once identified, changes in baseline metrics over time could act as biomarkers for the detection and monitoring of neurodevelopmental, neuropsychiatric, or neurodegenerative disorders (74–77). Brain changes associated with neurodegenerative disorders typically occur over years (64, 78), while neurodevelopmental disorders impact brain function across the lifespan (79, 80). Conversely, some disorders (e.g., Multiple Sclerosis, Lewy Body Dementia, Schizophrenia) are also characterized by fluctuating cognitive symptoms at the scale of days or week (81–86). Consequently, cognitive biomarkers are likely to be more effective when brain activity is intermittently monitored over long periods (11), or in intensive bursts of frequent sampling (87).

This study had some limitations. The ideal comparison between dry vs. wet EEG would involve near-daily at-home wet EEG, but the burden of this design renders this unfeasible as wet EEG requires the presence of a trained technician for set-up. Some brain data variability could be related to task learning effects (both in specific task performance—see Supplementary Figure S11—and usability of the technology) rather than device performance. In addition, it is also likely that participants improved at using the platform during the first week of recordings. To quantify the amount of variability emerging from learning how to use the headset itself, future studies should consider first providing participants with the tablet before introducing the headset at a later stage. The headset correct placement in the home was not controlled and may have introduced noise in the signals. However, the risk of misplacement was mitigated using a semi-rigid neoprene frame which ensured the correct relative electrodes positions. The headset has also natural orientation points to ensure a consistent correct positioning: the earpieces go behind the ears and the front strap aligns with the brows. Specific emphasis was also given to participants during onboarding on the importance of headset placement and reminders were sent at the beginning of each session *via* the mobile app on the procedure to ensure a correct use. However, future studies may consider evaluating this specific question by taking pictures of participants in the home. Part of the signal variability may also originate from day-to-day changes related to lifestyle factors, mood, and stress levels, which we did not quantify here. Different preprocessing pipelines were applied in our studies and the reference dataset, and it is possible that further optimization of data exclusion strategies could result in improved SME levels for the wet lab and/or dry home data. The younger cohort dataset did not include any females, preventing us from evaluating the impact of sex on the variability of the data. Additionally, future studies will have to test validity in specific target clinical populations for whom it may be relevant to increase stimulus size and presentation time. Finally, the studies differed on numerous levels including site, age, gender, educational background, motivation for participation, task protocol, cadence of sessions, and number of in-lab sessions, which prevented us from performing direct comparison.

## Conclusions

Overall, our study contributed to the field in the following ways. We evaluated two innovative neurocognitive studies with repeated sampling in a remote setting, conducted with cohorts of younger and older age profiles. Participants regularly and successfully used a portable dry EEG system and recorded behavioral and ERP data comparable to traditional wet EEG paradigms. This generated rich longitudinal data that would not have been possible in a traditional experimental laboratory-based design. Due to the volume of data collected (i.e., near daily), multiple strategies were employed to improve signal quality, including aggregation of multiple sessions within subjects and conservative data inclusion protocols. ERP data were similar in morphology to those reported in the literature from laboratory-based studies, and with aggregation, signal quality was also comparable. Overall, these results demonstrate that portable EEG technology is a suitable tool for cognitive neuroscience investigations, and has the potential to provide objective, frequent and patient-centered tracking of biomarkers of functional neurophysiology. This approach has potential to facilitate large scale longitudinal studies of neurodegenerative, neurodevelopmental and neuropsychiatric disorders that manifest on different time scales.

## Data availability statement

The datasets presented in this article are not readily available because the data collected using the Cumulus platform is commercially sensitive and contains proprietary information. Requests to access supporting data will be considered from bona fide researchers upon reasonable request. Requests to access the datasets should be directed to florentine@cumulusneuro.com.

## Ethics statement

The studies involving human participants were reviewed and approved by Institutional Review Board of the Otto-von-Guericke University Magdeburg, Germany and the Ethics Committee of the School of Psychology of Trinity College Dublin, Ireland. The patients/participants provided their written informed consent to participate in this study.

## Author contributions

FB: conceptualization, methodology, software, formal analysis, investigation, data curation, writing—original draft, writing—review and editing, visualization, and project administration. FF: conceptualization, methodology, project administration, and writing—review and editing. AB: conceptualization, methodology, investigation, writing—review and editing, and project administration. LD: conceptualization, methodology, writing—review and editing, and project administration. JD: conceptualization, methodology, and writing—review and editing. MI and LR-D: software and writing—review and editing. MK: conceptualization, methodology, writing—review and editing, and project administration. BM: conceptualization, methodology, supervision, and writing—review and editing. HN: writing—review and editing, software, and data curation. MW: conceptualization, methodology, and supervision. RW: conceptualization, methodology, supervision, and writing—review and editing. All authors contributed to the article and approved the submitted version.

## Funding

The studies on which this paper is based were funded by the Irish Research Council (IRC), Science Foundation Ireland and Cumulus Neuroscience Ltd. FB was supported by the IRC Employment Based Postgraduate Programme (EBPPG/2019/53), FF by the IRC Enterprise Partnership Postdoctoral Scheme (EPSPD/2017/110). LR-D was supported by the Science Foundation Ireland (18/IF/6272). LD was funded by the Leibniz Institute for Neurobiology in Magdeburg, Germany (SFB779). MK was supported by the Jena University Hospital. FB and FF received funding from Cumulus Neuroscience Ltd., in partnership the Irish Research Council, through the IRC Employment Based Postgraduate Programme (EBPPG/2019/53), and the IRC Enterprise Partnership Postdoctoral Scheme (EPSPD/2017/110), respectively, with RW as the mentor on both these projects.

## Conflict of interest

Conflict of interest FB, AB, JD, MI, BM, HN, and LR-D are employees of Cumulus Neuroscience Ltd., a company that develops and provides dry EEG technology. The remaining authors declare that the research was conducted in the absence of any commercial or financial relationships that could be construed as a potential conflict of interest.

## Publisher's note

All claims expressed in this article are solely those of the authors and do not necessarily represent those of their affiliated organizations, or those of the publisher, the editors and the reviewers. Any product that may be evaluated in this article, or claim that may be made by its manufacturer, is not guaranteed or endorsed by the publisher.
