# Supplementary material for: Neuroscience from the comfort of your home: Repeated, self-administered wireless dry EEG measures brain function with high fidelity
Source: Front Digit Health. 2022 Jul 29;4:944753. doi: 10.3389/fdgth.2022.944753 (PMC9372279; doi:10.3389/fdgth.2022.944753)
Supplement: Supplementary file 1 [file Data_Sheet_1.DOCX]

Neuroscience from the comfort of your home: repeated, self-administered wireless dry EEG measures brain function with high fidelity

# Supplemental materials

**Table S1: Count of usable sessions per channel.** ^a^Only data collected before the pharmacological interventions are evaluated here, so figures may vary somewhat relative to the figures cited in section 3.2.

| Channel | *Oddball Task - Older Adults (N=50)* | *Oddball Task - Younger Adults^a^ (N=30)* | *Flanker Task - Older Adults (N=50)* | *Flanker Task - Younger Adults^a^ (N=30)* |
| --- | --- | --- | --- | --- |
| O1 | 495 | 292 | 466 | 297 |
| O2 | 505 | 286 | 482 | 285 |
| Pz | 634 | 339 | 593 | 343 |
| P3 | 665 | 342 | 603 | 343 |
| CPz | 617 | 323 | 578 | 323 |
| P4 | 610 | 337 | 572 | 335 |
| Cz | 574 | 313 | 541 | 316 |
| FC3 | 647 | 338 | 603 | 341 |
| FCz | 619 | 337 | 577 | 339 |
| FC4 | 631 | 331 | 595 | 334 |
| Fz | 643 | 329 | 592 | 332 |
| FT7 | 650 | 334 | 610 | 336 |
| AF7 | 646 | 335 | 589 | 335 |
| Fpz | 645 | 343 | 592 | 346 |
| AF8 | 649 | 337 | 602 | 342 |
| FT8 | 608 | 314 | 570 | 314 |
| Average | **614.88** | **326.88** | **572.81** | **328.81** |
| Std Err. | **49.96** | **17.25** | **42.17** | **17.58** |
| Min | **495** | **286** | **466** | **285** |
| Max | **665** | **343** | **610** | **346** |

**Table S2: Statistical Analyses of the System Usability Scale.** One-sided non-parametric Mann-Whitney U rank test were used to compare the two groups.

|  | **Older Adults (N=32)** | | **Younger Adults (N=18)** | |  |  |
| --- | --- | --- | --- | --- | --- | --- |
| **System Usability Scale** | ***Mean score (SD)*** | ***Mann–Whitney U statistic*** | ***Mean score (SD)*** | ***Mann–Whitney U statistic*** | ***Uncorrected p-values*** |  |
|  |  |  |  |  |  |  |
| *"I think I would like to use this system frequently"* | 3.47 (1.29) | 334.5 | 3.17 (0.99) | 241.5 | 0.8366 |  |
| *"I found the system unnecessarily complex"* | 1.69 (1.06) | 295.5 | 1.56 (0.78) | 280.5 | 0.4359 |  |
| *"I thought the system was easy to use"* | 4.62 (0.61) | 323 | 4.5 (0.62) | 253 | 0.8026 |  |
| *"I think that I would need the support of a technical person to be able to use this system"* | 1.28 (0.77) | 288 | 1.17 (0.38) | 288 | 0.5063 |  |
| *"I found the various functions in the system were well integrated"* | 4.53 (0.76) | 331.5 | 4.28 (1.02) | 244.5 | 0.8466 |  |
| *"I thought there was too much inconsistency in this system"* | 1.34 (0.6) | 180.5 | 1.94 (0.94) | 395.5 | 0.994 |  |
|  |  |  |  |  |  |  |
| *"I would imagine that most people would learn very quickly"* | 4.28 (0.85) | 313 | 4.28 (0.46) | 263 | 0.714 |  |
|  |  |  |  |  |  |  |
| *"I found the system very cumbersome to use"* | 1.59 (1.01) | 298.5 | 1.5 (0.99) | 277.5 | 0.405 |  |
|  |  |  |  |  |  |  |
| *"I felt confident using this system"* | 4.66 (0.83) | 300 | 4.72 (0.46) | 276 | 0.633 |  |
|  |  |  |  |  |  |  |
| *"I needed to learn a lot of things before I could get going with this system"* | 1.84 (1.05) | 357.5 | 1.44 (0.78) | 218.5 | 0.06 |  |
|  |  |  |  |  |  |  |
| **Composite Score** | 84.53 (10.15) | 303 | 83.33 (10.18) | 273 | 0.6235 |  |


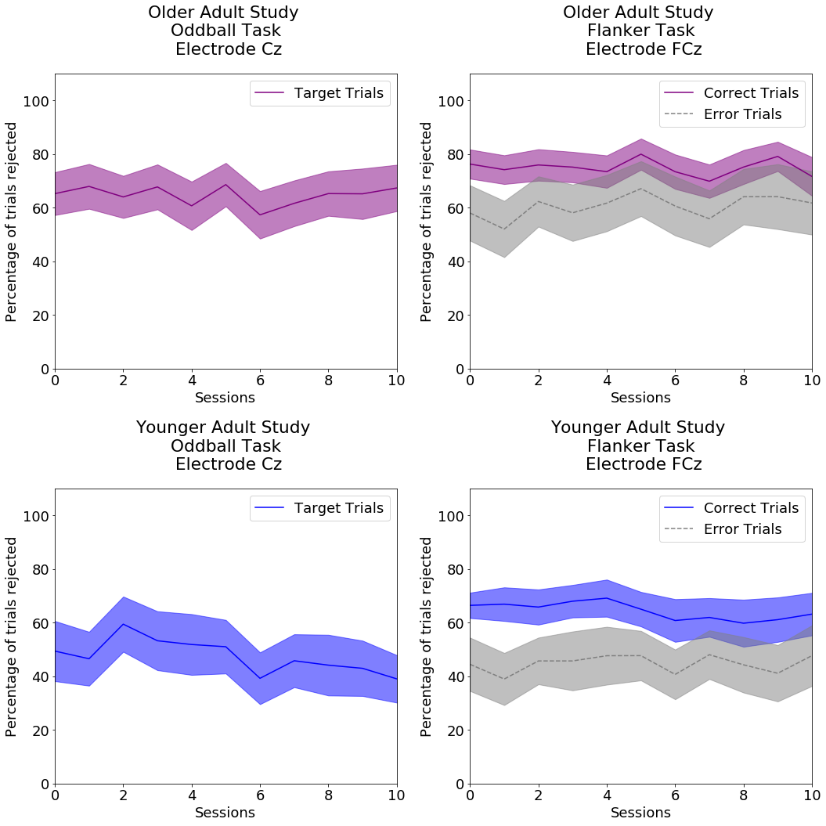


**Figure S1: Mean percentage of trials rejected across time.** Top row: Older Adult Study. Bottom Row: Younger Adult Study. Left Column: Target Trials extracted from the Oddball task. Right Column: Correct and Incorrect trials extracted from the Flanker tasks. Shaded areas correspond to the 95% confidence intervals.


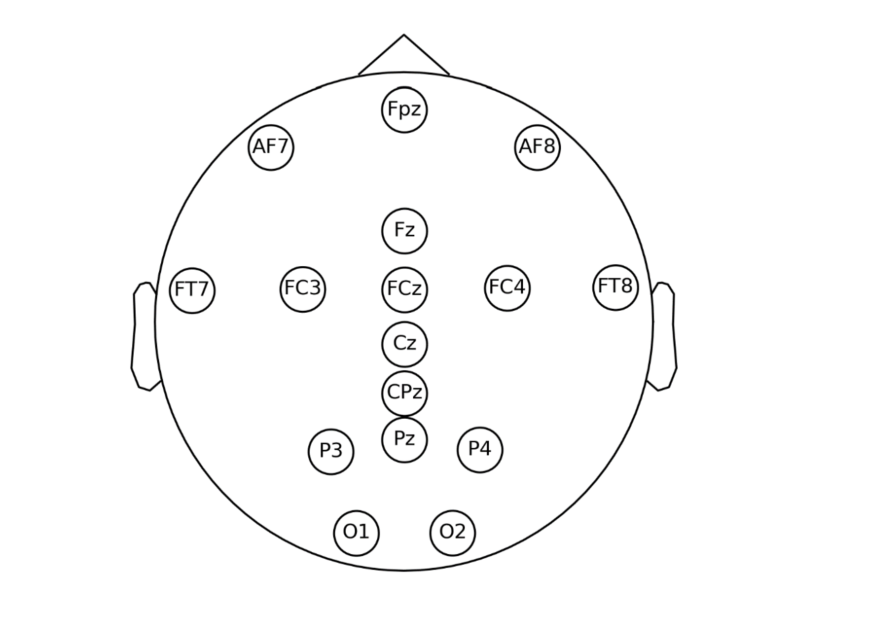


**Figure S2: Cumulus dry EEG recording headset scheme.** The Cumulus headset has 16 channels at standard 10-20 positions. Electrode labels encode the brain region (Anterior, Frontal, Temporal, Central, Parietal, Occipital) and hemisphere (odd numbers to the left, even to the right).


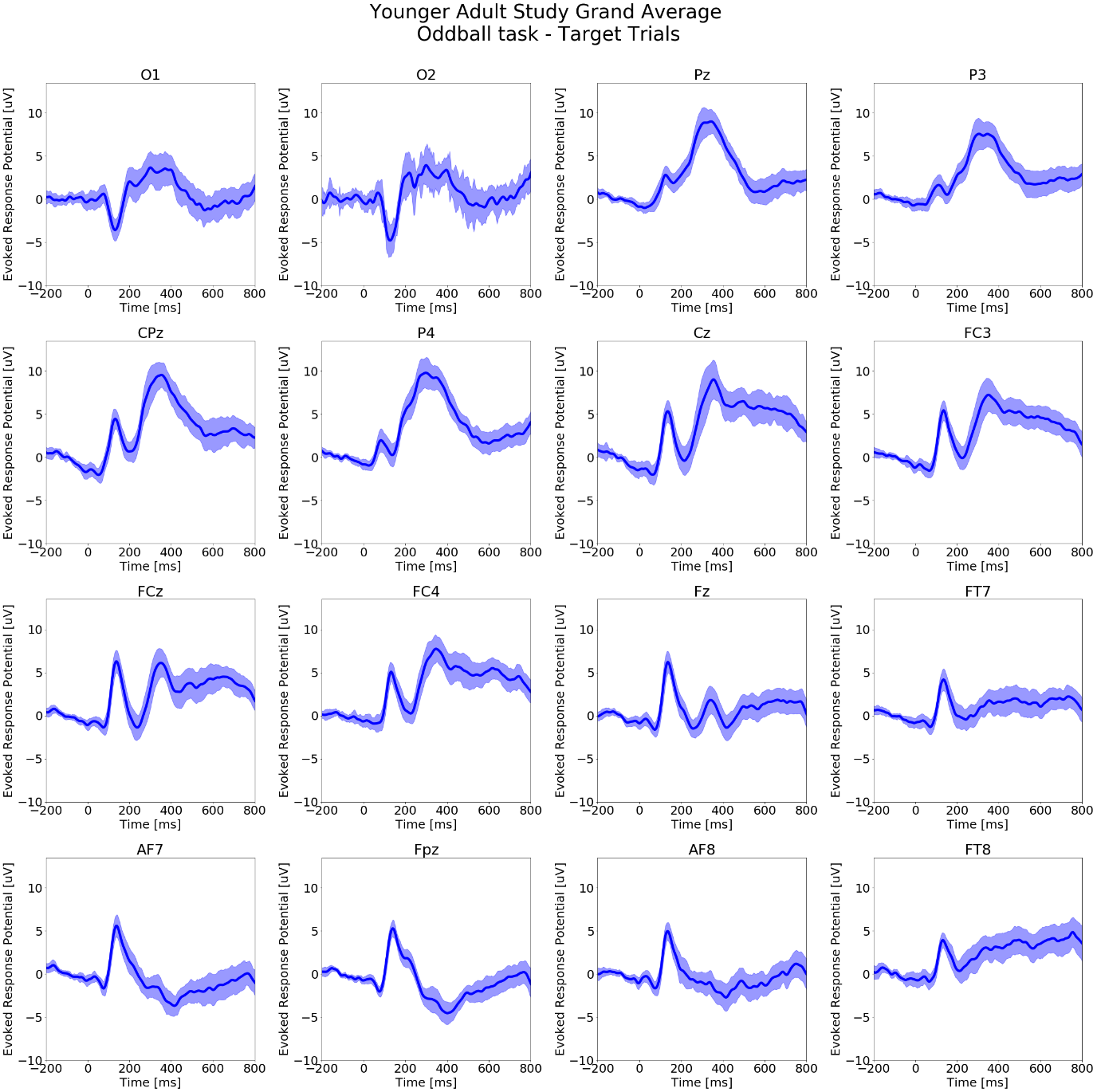


**Figure S3: Event-related potentials collected during the Younger Adult Study extracted from the Oddball task locked to the Target stimulus presentation.** From the top left plot to the bottom right plots: O1, O2, Pz, P3, CPz, P4 Cz, FC3, FCz, FC4, Fz, FT7, AF7, Fpz, AF8, and FT8. Shaded areas correspond to the 95% confidence intervals.


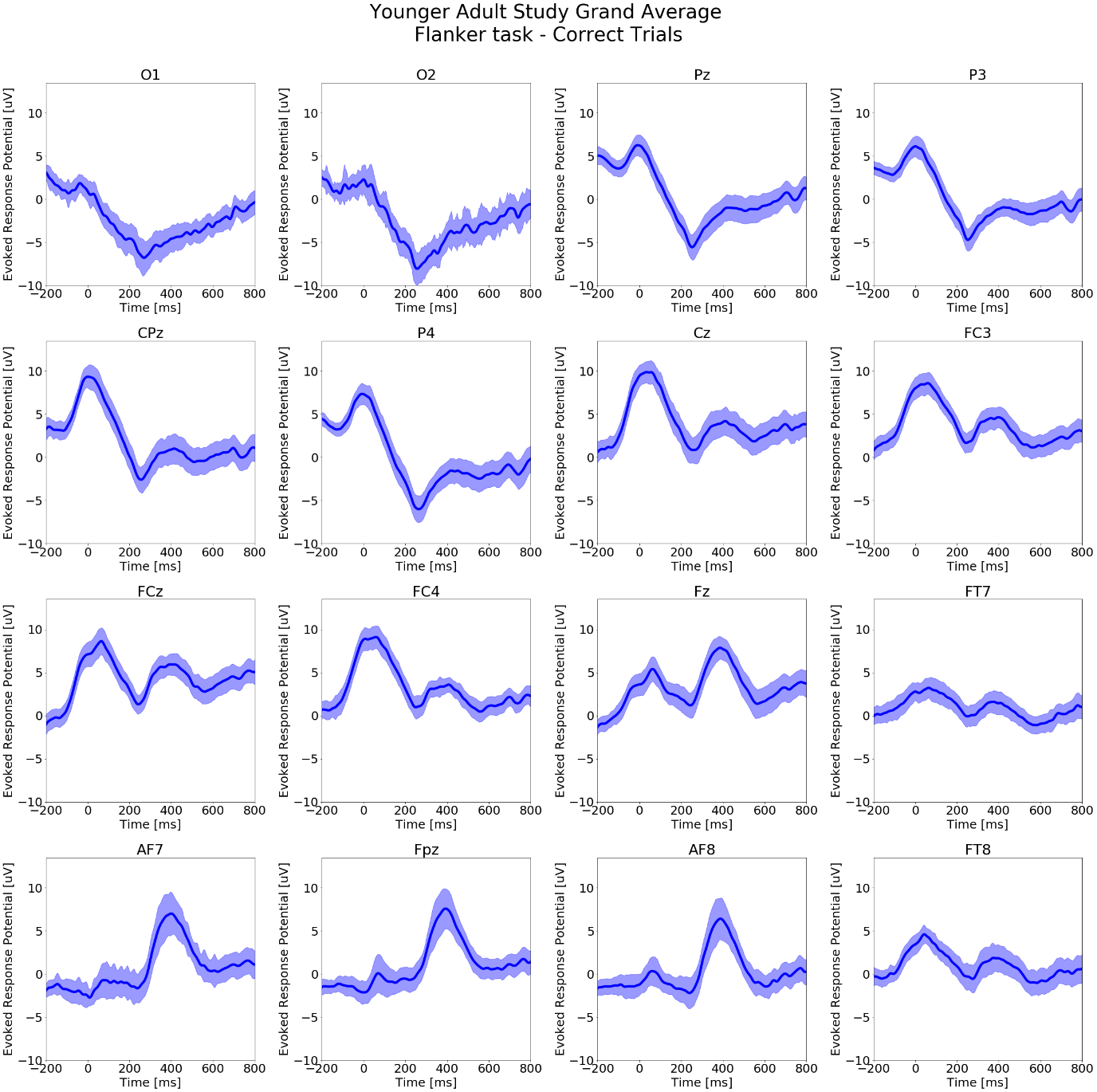


**Figure S4: Event-related potentials collected during the Younger Adult Study extracted from the Flanker task locked to participants’ response to the Correct Trials.** From the top left plot to the bottom right plots: O1, O2, Pz, P3, CPz, P4 Cz, FC3, FCz, FC4, Fz, FT7, AF7, Fpz, AF8, and FT8. Shaded areas correspond to the 95% confidence intervals.


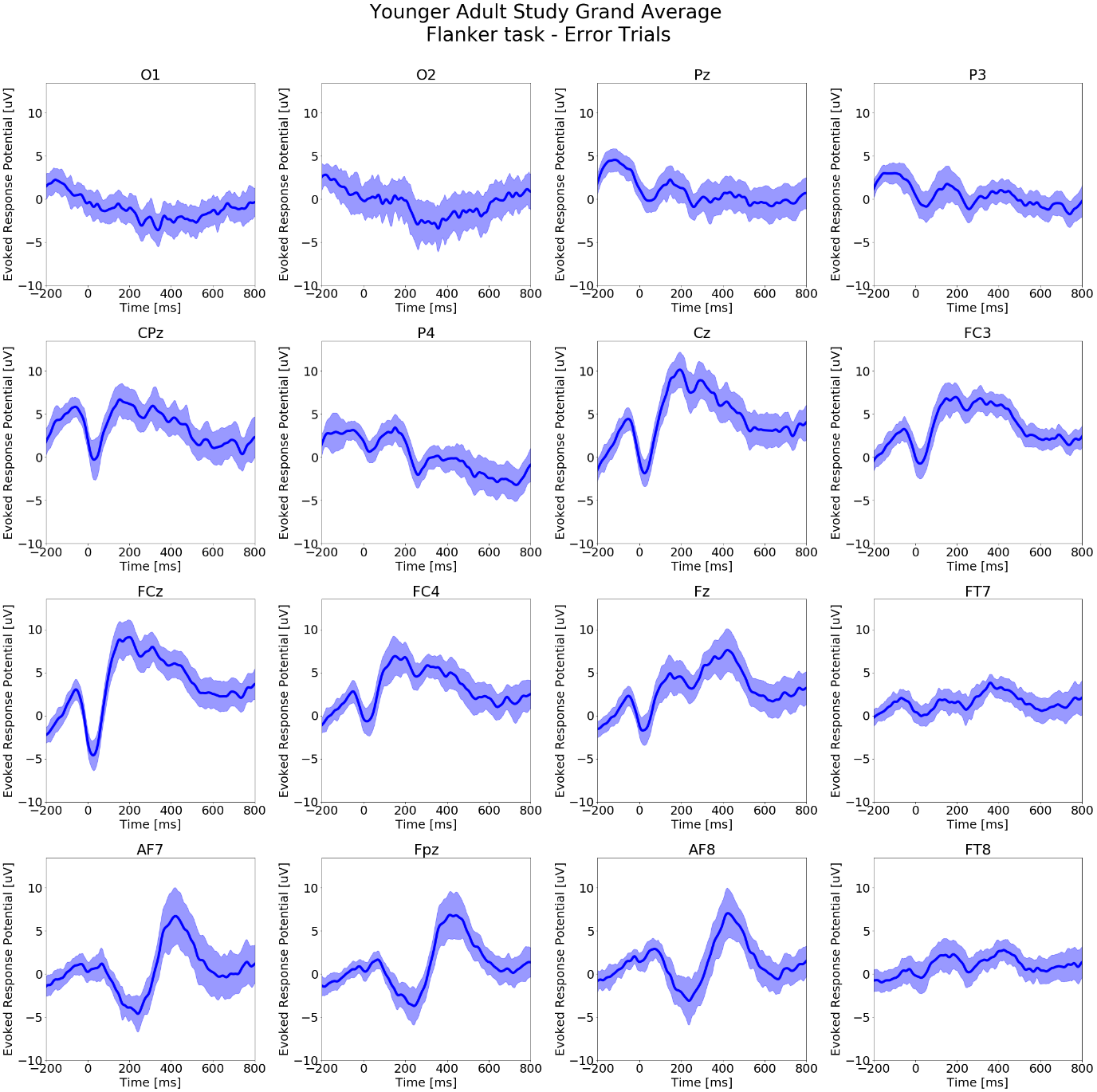


**Figure S5: Event-related potentials collected during the Younger Adult Study extracted from the Flanker task locked to participants’ response to the Correct Trials.** From the top left plot to the bottom right plots: O1, O2, Pz, P3, CPz, P4 Cz, FC3, FCz, FC4, Fz, FT7, AF7, Fpz, AF8, and FT8. Shaded areas correspond to the 95% confidence intervals.


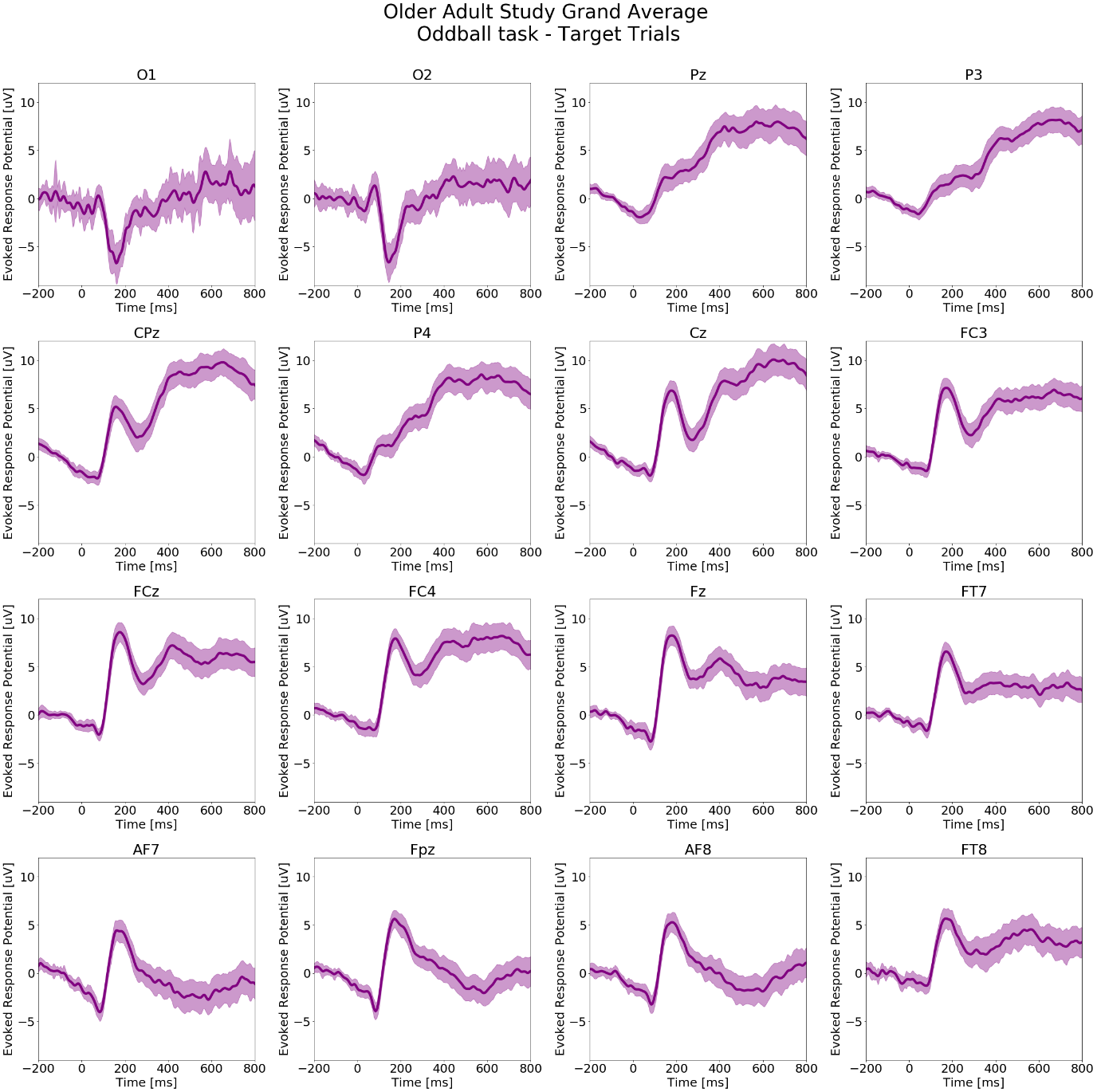


**Figure S6: Event-related potentials collected during the Older Adult Study extracted from the Oddball task locked to the Target stimulus presentation**. From the top left plot to the bottom right plots: O1, O2, Pz, P3, CPz, P4 Cz, FC3, FCz, FC4, Fz, FT7, AF7, Fpz, AF8, and FT8. Shaded areas correspond to the 95% confidence intervals.


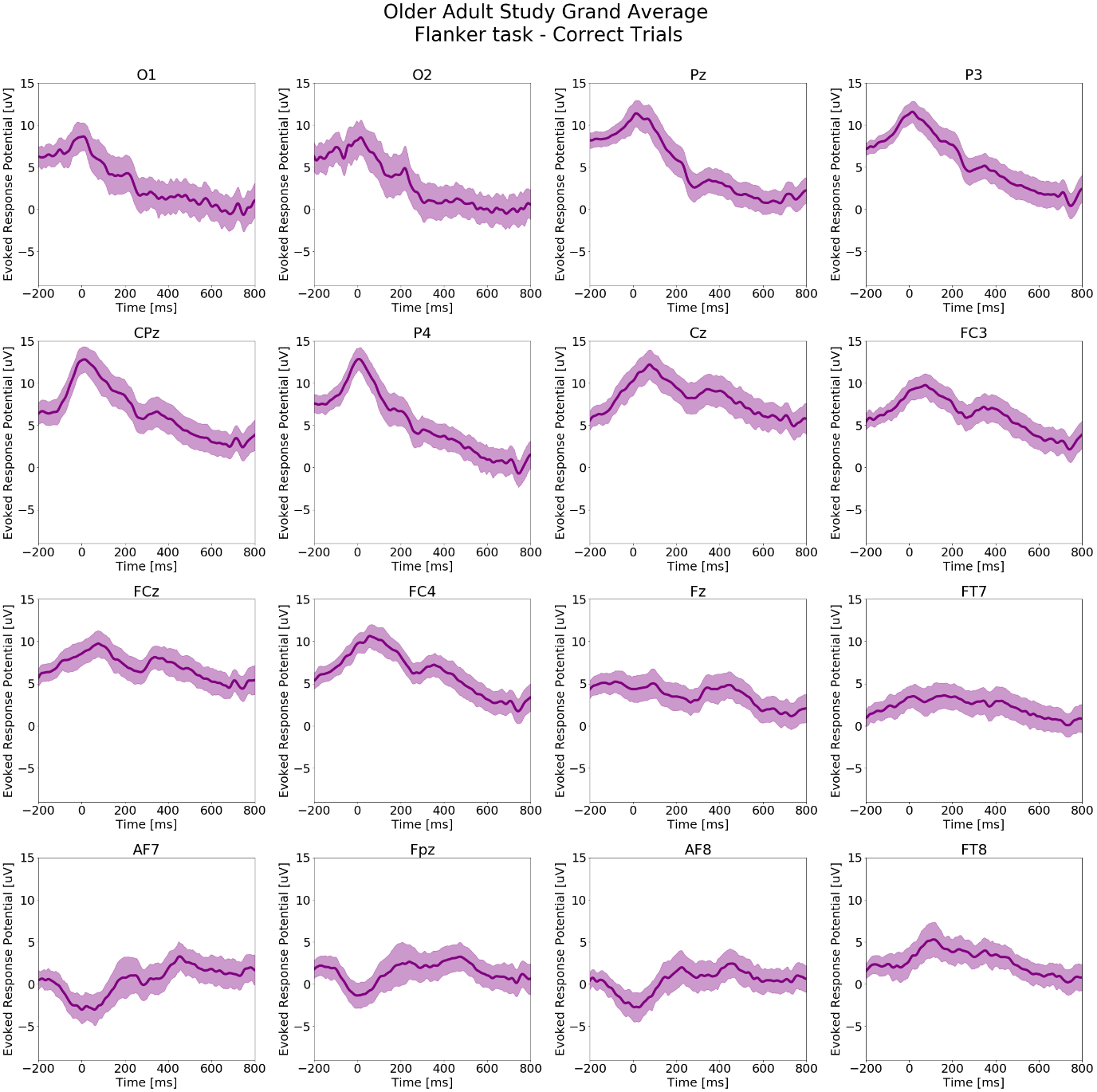


**Figure S7: Event-related potentials collected during the Older Adult Study extracted from the Flanker task locked to participants’ response to the Correct Trials.** From the top left plot to the bottom right plots: O1, O2, Pz, P3, CPz, P4 Cz, FC3, FCz, FC4, Fz, FT7, AF7, Fpz, AF8, and FT8. Shaded areas correspond to the 95% confidence intervals.


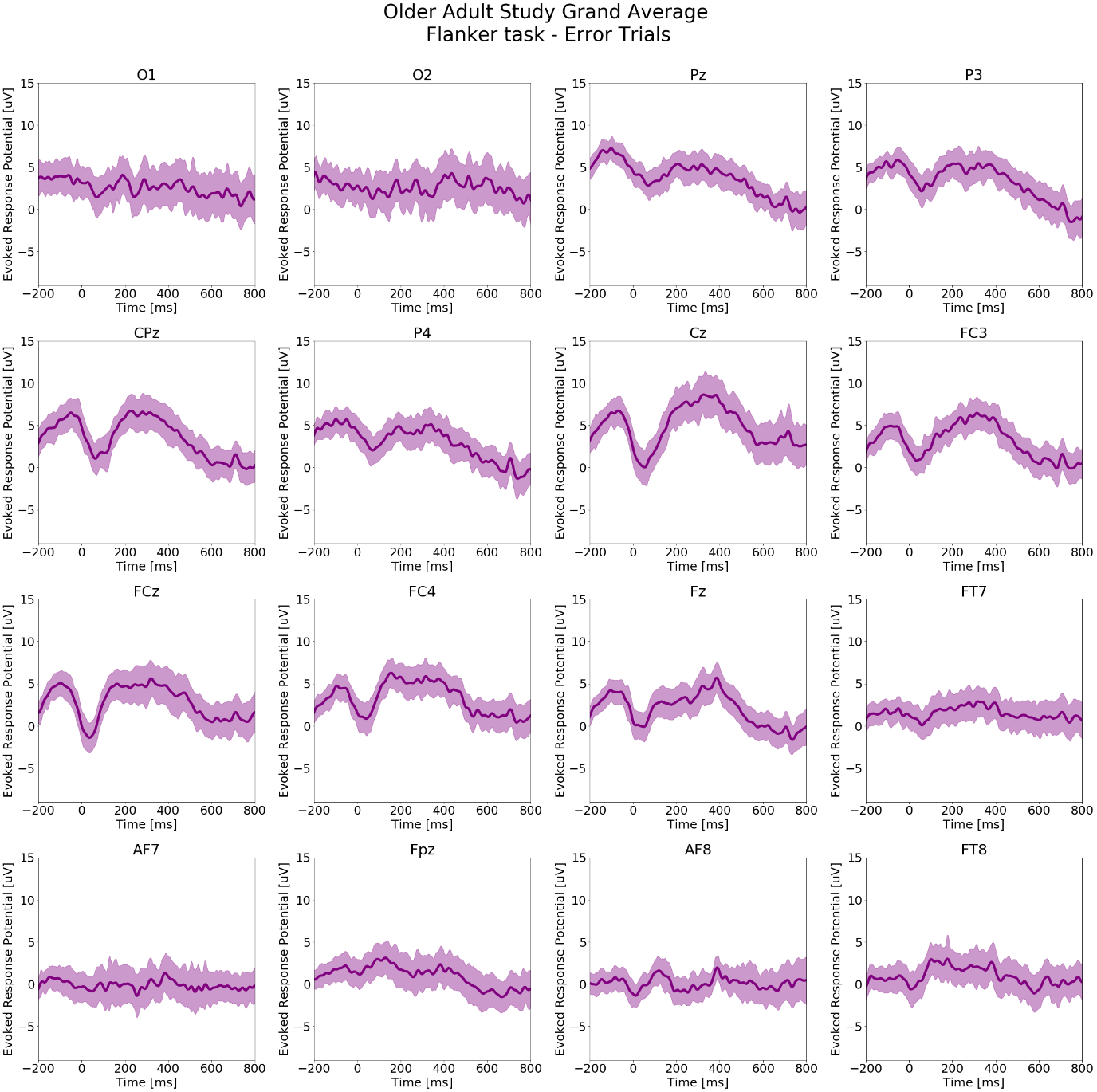


**Figure S8: Event-related potentials collected during the Older Adult Study extracted from the Flanker task locked to participants’ response to the Error Trials.** From the top left plot to the bottom right plots: O1, O2, Pz, P3, CPz, P4 Cz, FC3, FCz, FC4, Fz, FT7, AF7, Fpz, AF8, and FT8. Shaded areas correspond to the 95% confidence intervals.

# **Supplemental data analysis**

## Behavioral data analyses - Methods

Only behavioral data corresponding to correct trials are presented in this analysis. For the Oddball task, ‘correct trials’ refers to correct target identification. For the Flanker task, it corresponded to trials on which the target stimulus direction was correctly identified. To investigate the impact of practice effects on reaction time (RT), we computed participants’ mean RT across their first 7 sessions. This time window was selected so to avoid any confounding drug effects in the Younger Adult Study. For the Older Adult Study, only participants that completed at least 7 sessions of each task were included in these analyses. For the Younger Adult Study, only participants that completed at least 7 sessions of each task before the first infusion session were included in the analyses. Linear mixed-effects (LME) models were used to investigate the effect of sessions on RTs. In these models, participants were random intercepts and sessions were fixed effects. This analysis was conducted using the Statsmodels package (version 0.10.1; <https://www.statsmodels.org/devel/mixed_linear.html>, RRID:SCR_016074) in Python (version 3.6.9, RRID:SCR_008394). Normality of the residuals distribution was assessed by visual inspection.

## Behavioral data analyses - Results

In the Older Adult Study, the LME model to analyze RT in the Oddball task contained 343 observations. The LME model to analyze Flanker task RT in all successful trials, and in RT differences in successful incongruent minus congruent trials, contained 336 observations. There was a significant effect of session on RT in the Oddball task, with an average estimated RT decrease of 22.87 ms (SE=1.54, p<10e-49, df_residuals_=341) on each session. There was also a significant effect of sessions on RT in the Flanker task for all successful trials, with an average estimated RT decrease of 17.88 ms (SE=1.01, p<10e-69, df_residuals_=334) on each session. The difference in RT between successful incongruent and congruent flanker trials was also significant, with an average estimated decrease of differences of 3.03 ms (SE=0.52, p<10e-9, df_residuals_=334) between the two RTs with each session. Full results are reported in S11 and Figure S10

In the Younger Adult Study, the LME model analyzing Oddball task RT contained 152 observations. There was a significant effect of sessions on RT, with an average estimated RT decrease of 7.24 ms (SE=1.11, p<10e-10, df_residuals_=150) on each session. The LME model analyzing Flanker task RT in all successful trials, and differences in RT in successful incongruent minus congruent trials, contained 138 observations. There was a significant effect of sessions on RT with an average estimated RT decrease of 8.87 ms (SE=0.9, p<10e-22, df_residuals_=136) on each session. The effect of session over the difference in RT across incongruent minus congruent trials was not significant. The decrease of difference between the two RTs by session was estimated at 1.05 ms (SE=0.54, p=0.050, df_residuals_=136). Full results are reported in Table S11 and Figure S10.


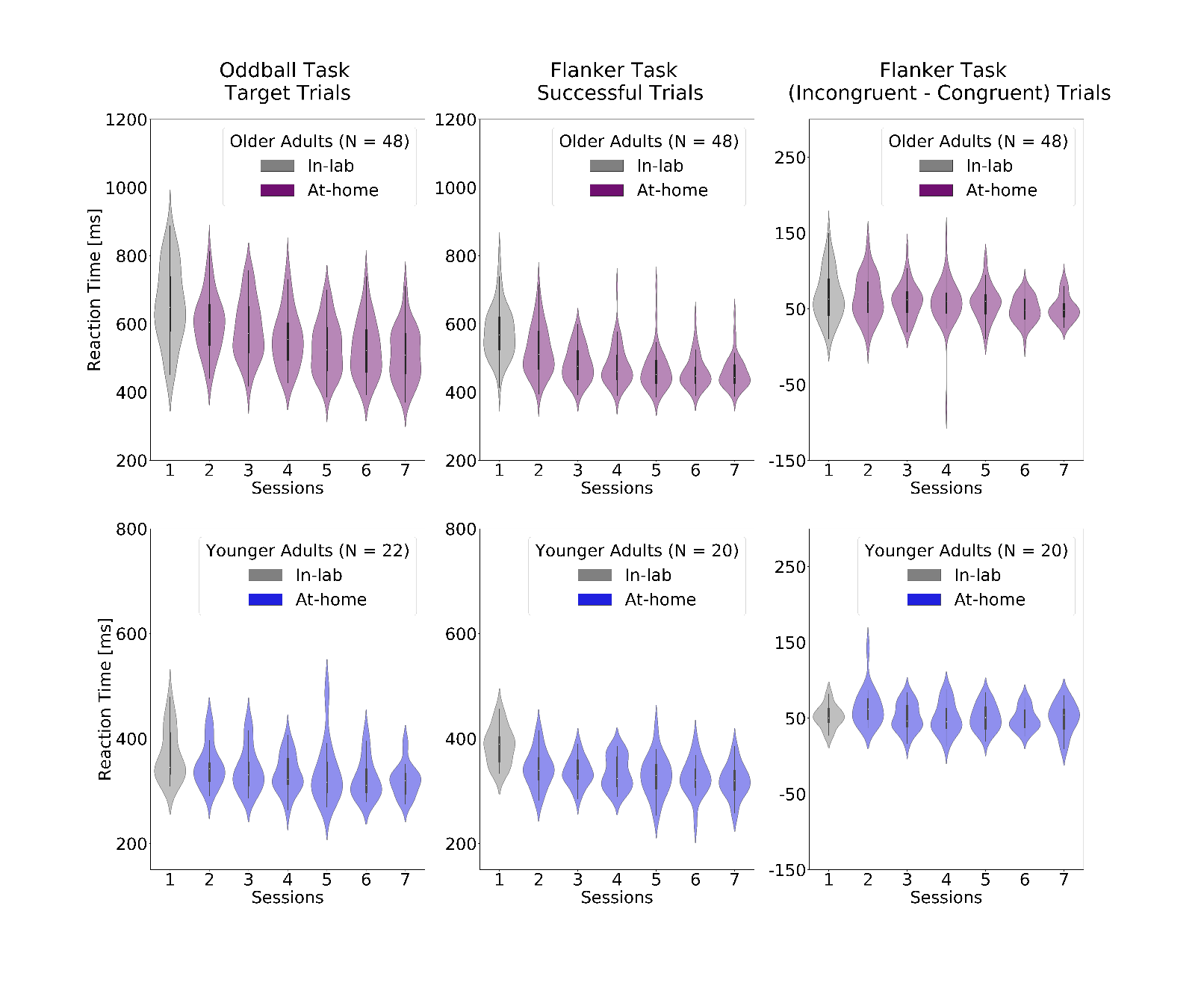


**Figure S9: Behavioral responses to the gamified tasks during the first seven sessions** in the Data of the Older Adult Study are depicted in purple (top row) and of the Younger Adult Study in blue (bottom row). From left to right: RT to the successful trials in the gamified Oddball task RT to the successful trials in the gamified Flanker task, difference in RT between the incongruent minus congruent successful trials in the gamified Flanker task (N older adults = 48, N younger adults = 20).

**Table S10: Linear mixed effect model estimates and Wald tests of behavioral data.** Estimates of the ﬁxed and random effects with their corresponding standard error (SE) and statistics of the individual estimates are provided.

|  | Task and  Behavioral Metric | Coef. (SE) | | z-stat | P-values | 95% Confidence Interval | Residual DFs |
| --- | --- | --- | --- | --- | --- | --- | --- |
| Older Adult Study | Oddball  RT | Intercept | 658.92 (11.95) | 55.16 | <software resolution | [635.02, 682.33] | 341 |
|  |  | Slope | -22.87 (1.54) | -14.83 | <10e-49 | [-25.89, - 19.85] |  |
|  | Flanker  RT  Successful trials | Intercept | 562.36 (8.46) | 66.49 | <software resolution | [545.78, 578.93] | 334 |
|  |  | Slope | -17.89 (1.01) | -17.65 | <10e-69 | [-19.87, - 15.90] |  |
|  | Flanker  Difference RT (Incongruent-Congruent) trials | Intercept | 70.47 (3.28) | 21.46 | <10e-101 | [64.03, 76.9] | 334 |
|  |  | Slope | -3.03 (0.52) | -5.79 | <10e-8 | [-4.05, -2.00] |  |
| Younger Adult Study | Oddball  RT | Intercept | 367.6 (8.56) | 42.94 | <software resolution | [350.82, 384.38] | 150 |
|  |  | Slope | -7.24 (1.11) | -6.5 | <10e-10 | [-9.42, -5.06] |  |
|  | Flanker  RT  Successful trials | Intercept | 374.89 (7.46) | 50.25 | <software resolution | [360.27, 389.51] | 136 |
|  |  | Slope | -8.87 (0.9) | -9.85 | <10e-22 | [-10.63, -7.10] |  |
|  | Flanker  Difference RT (Incongruent-Congruent) trials | Intercept | 56.89 (4.10) | 13.87 | <10e-43 | [48.86, 64.93] | 136 |
|  |  | Slope | -1.05 (0.54) | -1.96 | 0.050 | [-2.1, 0.001] |  |
